# Antibodies and cryptographic hash functions: quantifying the specificity paradox

**DOI:** 10.3389/fimmu.2025.1585421

**Published:** 2025-11-05

**Authors:** Robert J. Petrella

**Affiliations:** 1 Department of Chemistry and Chemical Biology, Harvard University, Cambridge, MA, United States; 2 Harvard Medical School, Boston, MA, United States

**Keywords:** antibodies, adaptive immune system, receptors, antigens, epitopes, degeneracy, polyspecificity, polyreactivity

## Abstract

The specificity of the immune response is critical to its biological function, yet the generality of immune recognition implies that antibody binding is multispecific or degenerate. The current work explores and quantifies this paradox through a systems analysis approach that incorporates set theoretic ideas and an application of structural and statistical modeling to prior experimental immunological and biochemical data. Order-of-magnitude estimates are computed for the average degeneracies and specificities of antibodies and epitopes using a chemico-spatial model for epitope diversity and a binary model for antibody-antigen binding. The results illustrate and quantify how the humoral immune system achieves both high specificity and high degeneracy simultaneously by effectively decoupling the two properties, similarly to programs in cryptography called secure hash algorithms (SHAs), which display the same paradoxical features. In addition, an antibody-epitope interaction probability model is used to help show how newly formed antibodies may avoid cross-reactivity with self-antigens despite their high degree of multispecificity and how the requirement of polyclonal binding likely improves the overall specificity of the immune response. Because they describe the relationships between various statistical parameters in humoral immunity, the models developed here may also have predictive utility.

## 1 Introduction

Human antibodies (Abs) behave as specific to their cognate antigens (Ags) under many clinical and experimental conditions. For example, a monoclonal antibody’s specificity (1) is often critical to its therapeutic (2, 3) or diagnostic (4–6) utility. Such antibodies have commonly been referred to as “monospecific” or “monoreactive” (7–10). Early immunological thinking was, in fact, that one antibody or receptor implied one specificity (11, 12), in what has been referred to as the “one antibody, one antigen” dogma, rule or paradigm (13–15). The specificity of antibodies depends on the broad chemical and structural diversity in their variable or binding regions, which arises from a more-or-less random recombination of their coding immune gene segments (16, 17), together with several other secondary mechanisms (18–24).

Yet despite the large degree of diversity among immune cell receptors and antibodies, we know that their binding to antigens must still be highly multispecific, cross-reactive or *degenerate* (25–30). This is because immune recognition is thought to be inclusive of all types of antigen-sized molecules and molecular fragments (31–36)– an observation termed the postulate of *antigenic totality* in the present work– and while the immune repertoire of an individual is large, it is small compared to chemical space. In the language of set theory, the relation (“mapping”) of distinct antigens– or, more precisely, the parts of their structures called epitopes–to antibody species that can bind them must be many-to-one, at least on average.

The current work is an attempt to quantify and shed light on this specificity paradox. How can antibodies be both specific and multispecific? The topic has been discussed for decades with respect to both antibodies (37) and T-cells (28, 38), and estimates of T-cell receptor degeneracy have been given (25, 29). Sewell hypothesized that the capacity of T-cell receptors to retain some specificity for particular antigens despite high levels of cross-reactivity related to the sizes of their repertoires and those of their presenting peptides (28). With respect to antibodies, the current thinking is that they likely span a range of specificities, and that at least some antibodies produced late in the immune response are highly specific to their cognate antigens (39–41).

However, there has not been a formal, systematic attempt to describe the statistics of antibody-epitope interactions and to clarify–in mathematical terms–the paradoxical capacity of the adaptive immune response to display features of both multispecificity, or degeneracy, and specificity. The current study illustrates how these two properties are, in fact, distinct and statistically uncoupled. It does so by applying some set theoretic constructs and a quantitative though approximate (order-of-magnitude) systems analysis to the question. The study defines operational specificity (OpS) of antibodies precisely as how unlikely it is for an antibody to cross-react with an antigen that did not elicit it (i.e., a non-cognate antigen). It derives mathematical expressions for this quantity in regard to individual antibodies, their averages, and the antibody repertoire as a whole (systemic OpS), in terms of the other properties of the system. A binary, statistical model of antibody-antigen binding is developed (i.e., a pair either binds or it does not) and applied to prior experimental data to arrive at conservative, lower-bound estimates for antibody and epitope degeneracy, as well as cross-reactive probabilities and OpS. A related model (AEIP) is used to confirm the results and explore the frequency of antibody interaction with self-antigens, as well as the effect of polyclonality on self-interaction.

The main findings in the study are as follows:

1. A conservative, lower bound estimate for the average binding degeneracy of a human antibody is in the range of 10^73^ to 10^76^ epitopes, of which at least at least ≈10^18^ represent protein or peptide epitopes.
 To arrive at these estimates, a peptide-epitope chemico-spatial (PECS) model of epitope diversity is developed and combined with prior experimental data (Methods Section 2.1 and Results Sections 3.1 and 3.2).
2. An estimate for the average operational specificity (OpS) of human antibodies across a single individual’s antibody repertoire is approximately 1-10^–7^ to 1-10^-12^ (Results Section 3.3.1).
3. The systemic OpS–i.e. the specificity of an individual’s antibody repertoire as a whole, *S_c_*–varies as $S_{c}\approx 1-\frac{{\langle D_{i}\rangle }^{2}}{N}\left(\text{Var}\left(R_{j}\right)+1\right)$, where ⟨*D_i_*⟩ is the average epitope degeneracy, *R_j_* is the distribution of normalized antibody degeneracies, and *N* is the size of the repertoire. (Results Section 3.3.3 and **Appendix Section 6.2.3**).
4. Numerical estimates of human systemic antibody OpS are in the range of ≈1-10^–7^ to 1-10^–14^. (Results Section 3.3.3.)
5. The specificity of individual epitopes for their cognate antibodies is quite high: in the range of 1−10^−14^ to 1−10^−8^, but epitope space is so large that it virtually guarantees, statistically, that two randomly chosen antibodies in an immune repertoire will share many common epitopes in their binding spaces–conservatively, ≈ 10^6^ to 10^16^ protein or peptide epitopes, on average (Results Section 3.5), although this is a very small fraction of the total size of the relevant epitope space.
6. The average number of self-antigens to which a newly formed antibody will be complementary is in the range of $10^{-3}$ to 1, assuming 10,000 self-antigens and an average epitope degeneracy of 1 (see Results Section 3.7). This is consistent with experimental data.
7. The total number of antigens complementary to a polyclonal response of *n* antibodies increases approximately linearly with *n*, but the number of antigens having complementarity to multiple members (*m*) of that set of antibodies falls exponentially with *m*. (Results Section 3.8) This illustrates how the requirement of polyclonal binding in the immune response likely improves its overall specificity.

Further, it is illustrated here that the mathematical structure underlying immune specificity and degeneracy closely mirrors that of cryptographic hash functions (see ref (42) for review), also known as *secure hash algorithms* (SHAs). These functions take digital files as their input and generate relatively short alphanumeric codes called *hash values*, a.k.a. message digests, that are then attached to the files for security purposes. They are used in many types of digital security protocols, such as those generating digital signatures (43, 44). The Bitcoin mining protocol (45, 46) uses the hash algorithm SHA-256 (47, 48), which generates hash values of 256 bits in length. Mathematically, hash values and electronic files are the cryptographic counterparts of antibodies and epitopes, respectively, and they give rise to the same type of specificity paradox. Hash functions must be capable of handling any digital input, which means their outputs or digests must be highly degenerate (49), yet they must be specific enough to their originating or “cognate”^1^ files to ensure digital security. In addition, although an SHA is a total, single-valued function and the relation of epitopes to antibodies in a repertoire is not, we show that the latter approximates the former in behavior (see, e.g., Results Section 3.6). To illustrate the parallels between the systems, cryptanalytic data from a single case is compared to immunologic experimental data. The example case used is an electronic file that is 4000 bits (250 16-bit words) in size, which was approximately the size of the average Bitcoin transaction over most the 2010’s (50, 51).

By integrating experimental data into a newly developed mathematical framework that describes the relationships among key immune system properties or parameters, such as size and specificity, the present work aims to improve our understanding of the statistics of antibody-antigen complementarity. It shows that antibodies, at least on average, must have very high binding degeneracies or multispecificities and illustrates how they are able to maintain high clinical and laboratory specificity despite this. It further demonstrates how this capability relies on a statistical decoupling of specificity and multispecificity, similar to the case in cryptographic hash systems. The findings here also suggest that human immune system parameters have been evolutionarily optimized to permit universal antigen recognition while limiting cross- and self-reactivity. The study focuses on the statistics of humoral immunity–i.e., B-cell receptors and antibodies–but many of the general principles are applicable to T-cell receptors as well.

## 2 Methods

### 2.1 Peptide/protein epitope chemico-spatial model

**Figure 1:**
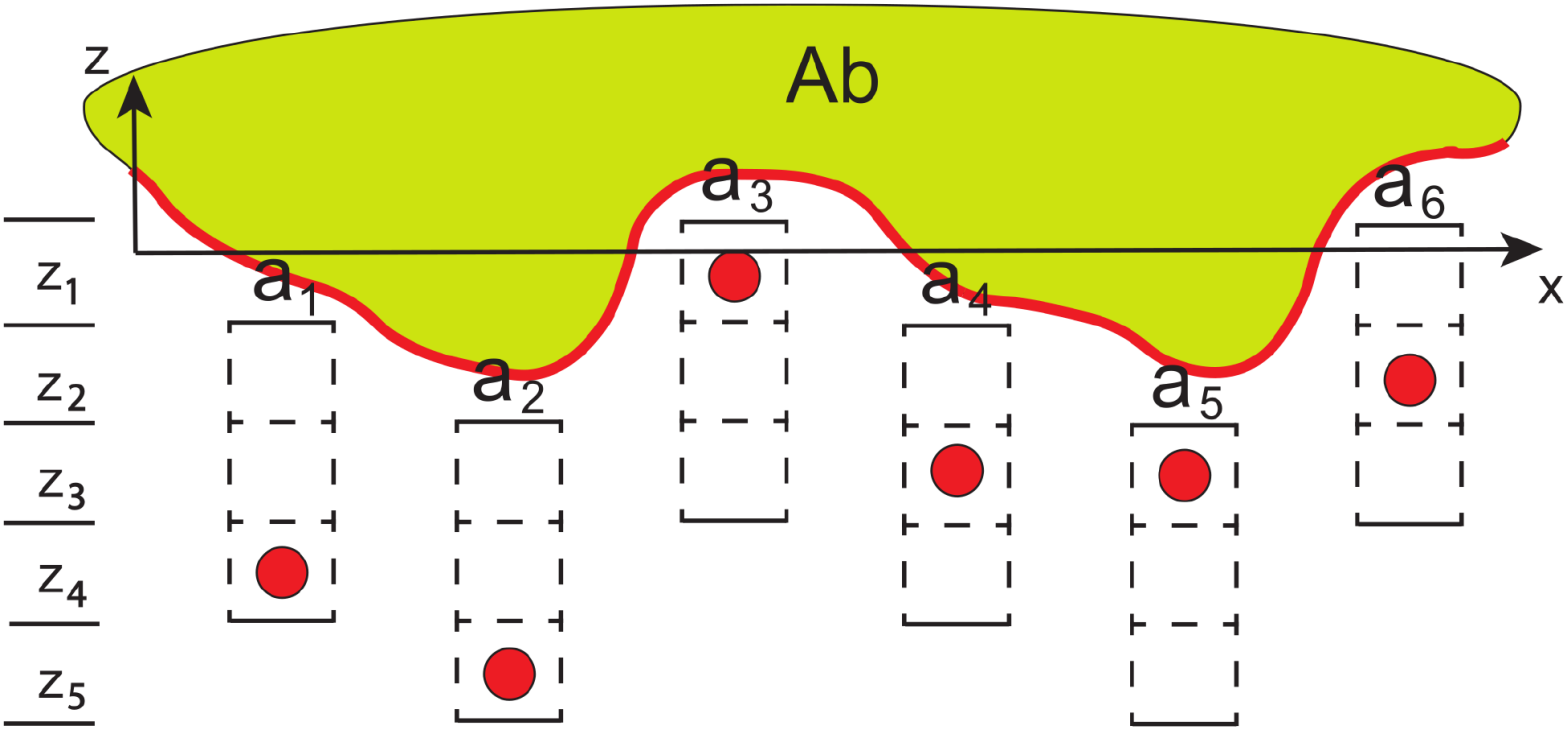
Peptide/protein epitope chemico-spatial (PECS) model. Ab–antibody; red border–region of antibody interfacing with epitopes; $a_{1}$ through $a_{6}$–6 possible (*x, y*)-positions for epitope amino acids; red discs–*α* carbons of amino acids; $z_{1}$ through $z_{5}$–5 possible *z*-positions for the *α*-carbons. The (hypothetical) interfacial plane, and additional $a_{k}$ positions, extend from the *x*-axis in the *y*-dimension, which would run perpendicular to the page. The *α* carbons can occur at any of three depths within each amino acid, and the local epitope surface can, itself, occur at one of three depths relative to the interfacial plane. The model intentionally undercounts the total number of epitopes by ignoring amino acid side chain and backbone conformational diversity, as well as possible shifts in amino acid $\left(a_{k}\right)$ position in the (*x, y*) plane.

We define an epitope here as *that portion of a molecular structure or set of structures (e.g., a set of amino acids) in a particular 3-D conformation, allowing for local fluctuations, that is involved in close interactions with an antibody.* (See Glossary in **Supplementary Material 2** for the definitions of terms used in this work.) Further, “epitopes” in this work generally refers to distinct epitopes, as opposed to copies, unless otherwise indicated.^2^ The size of epitope space depends not only on varying amino acid sequences, but also on conformational diversity, because antibodies can discriminate conformation (52, 53). Modeling this can be complex, but the approach is simplified here by use of a peptide/protein epitope chemico-spatial (PECS) model. In this model, each amino acid in a protein or peptide epitope can occur in any of *q* distinct (*x,y*) positions, where the hypothetical (*x,y*) plane is defined as roughly parallel to the paratope-epitope (Ab-Ag) interface. See **Figure 1**. In addition, each residue can occur at different depths, or *z*-positions, relative to the plane. The *z*-coordinate is decomposed into the position of the protein surface relative to the plane and that of the residue (as defined by its alpha-carbon) relative to the surface. The decomposition is important because the set of amino acids in an epitope is not necessarily continuous on the protein polypeptide chain (54). Most epitopes, in fact, are of the discontinuous or “conformational” type (55–59). This also suggests that the *z*-positions of the residues be considered as mutually independent. Hence, if *N_r_* amino acid types can occur at any one of *d* depths (*z*-positions) relative to the interfacial plane, the number of possible chemico-spatial configurations is $M_{prot}={\left(N_{r}d\right)}^{q}$. Because we are seeking a conservative, lower bound estimate for epitope diversity, the PECS model intentionally underestimates the total number of distinct protein/peptide epitopes (see also Appendix, Section 6.1).

### 2.2 Degeneracy and operational specificity

#### 2.2.1 Problem and solution element degeneracy

Consider finite sets Φ and Ψ containing *M* and *N* elements, respectively, and the relation

(1) H⊆Φ×Ψ.

We refer to Φ as the problem set, its elements *ϕ_i_* as problem elements, Ψ as the solution set, and its elements *ψ_j_* as solution elements. For simplicity and symmetry, throughout this work, the “*i*” subscripts–i.e., inputs–are reserved for problem elements and the “*j*” subscripts for solution elements.

**Figure 2:**
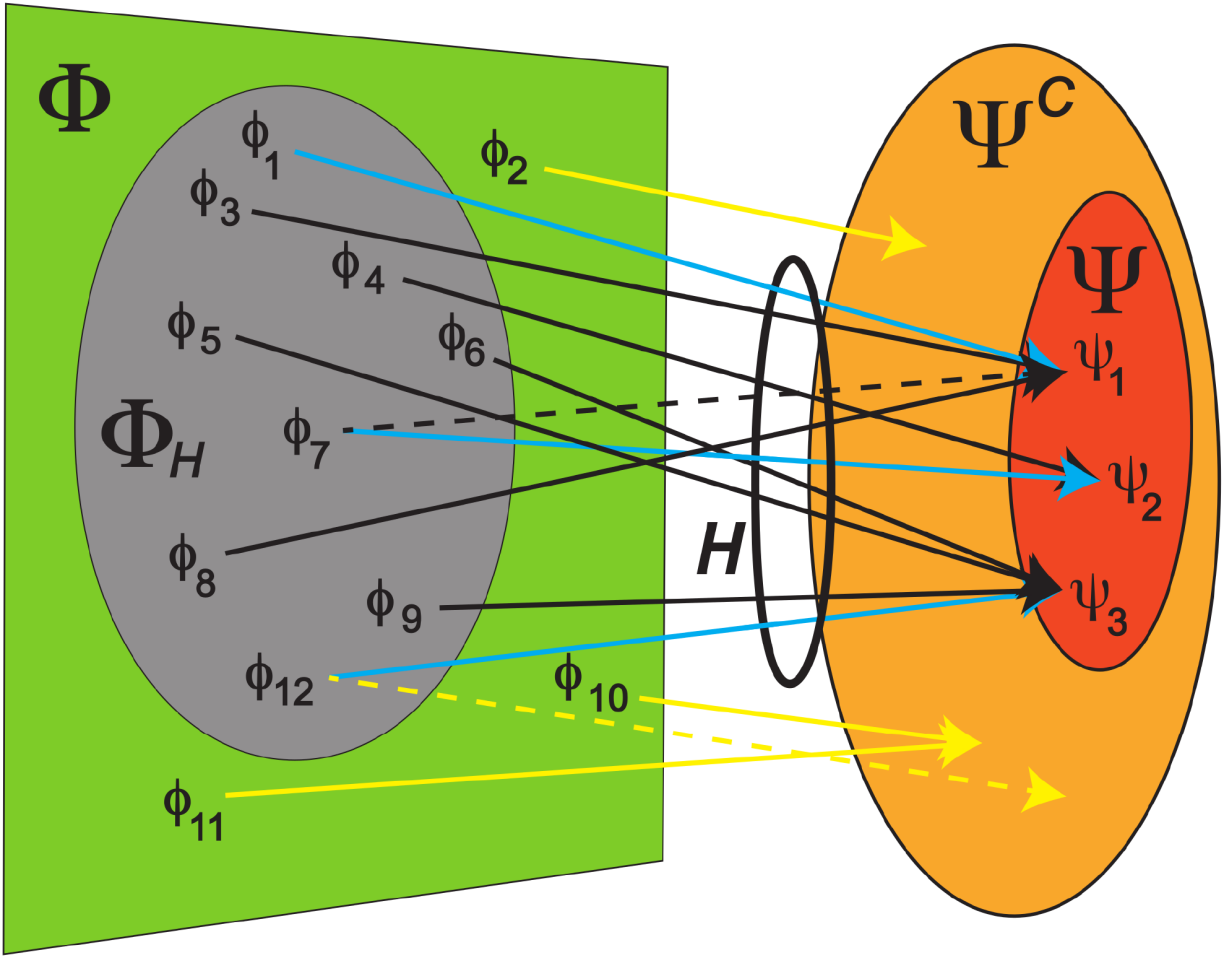
Diagram of relations associating problem and solution elements in the current study. Green trapezoid ( Φ)–the set of all epitopes (problem elements, domain), $\left\{\phi_{1},\phi_{2},\;\phi_{3},\ldots \phi_{12}\right\}$; red oval ( Ψ)–one individual’s Ab (variable region) repertoire (solution elements, codomain, image of H), $\left\{\psi_{1},\psi_{2},\psi_{3}\right\}$; H (ring)–the relation associating Φ and Ψ; grey oval ( ${\text{Φ}}_{\text{H}}$)–the subset of Φ that is related to Ψ by H–i.e., the preimage of H– which is the set of epitopes that bind to at least one Ab in Ψ; orange oval ( $\Psi^{\text{C}}$)–set of all possible human Ab species, of which Ψ is a subset; arrows–complementary $\left(\phi ,\psi^{\text{C}}\right)$ pairs, $\psi^{\text{C}}\in {\text{Ψ}}^{C}$; blue arrows–primary or cognate (ϕ,ψ) pairs; solid, black arrows–potential collisions, i.e., ( ϕ,ψ) pairs involving non-cognate ϕ; yellow arrows– $\left(\phi ,\psi^{\text{C}}\right)$ pairs involving Abs outside of Ψ; solid, yellow arrows–pairs involving epitopes that bind only to Abs outside of Ψ. The subset of problem elements, here $\left\{\phi_{1},\phi_{7},\phi_{12}\right\}$, involved in cognate pairs (blue arrows) is called ${\text{Φ}}_{cog}$ (see text). The dashed arrows represent pairs potentially involved in anticollisions: dashed, yellow–potential extra-repertoire anticollisions; dashed, black–a potential intra-repertoire anticollision ( $\psi_{1},\phi_{7},\psi_{2}$). The ( $\phi_{7},\psi_{1}$) pair also gives rise to a potential collision ( $\phi_{1},\psi_{1},\phi_{7}$). See text for the definitions of these variables in the cryptographic context. In both contexts, ${\text{Φ}}_{\text{H}}$ is many orders of magnitude larger than Ψ (not drawn to scale), and the H relation is presumed to be “onto”–i.e., covers the entire codomain Ψ.

As illustrated in **Figure 2**, Φ_H_ is the preimage of the *H* relation, Ψ is considered both the image and codomain of *H* and is embedded in a larger set of elements, Ψ^C^, the analysis of which is beyond the scope of the present study. In the immunologic context, Φ is the set of all possible epitopes, Ψ is the set of all antibodies in an individual’s repertoire, Φ_H_ is the set of epitopes that are complementary to (would bind to) at least 1 antibody (variable region) in Ψ, and Ψ^C^ is the set of all possible human antibodies (“Ψ complete”). In the cryptographic context, Φ and Ψ are the sets of all possible input files (in this work, of size 4000 bits) and all possible SHA-generated hash values (here, of 256 bits in length), respectively. Φ_H_ is the preimage of the SHA function, which is equal to Φ, and Ψ^C^ is the space of possible hash values producible by any SHA function. In both contexts, we assume that *H* is surjective–in other words, there is no need to consider the subset of Ψ called Ψ_H_ because all codomain elements (antibodies, hash values) are involved in the relation.^3^

For convenience, we define the relations *H_B_* and *H_K_* as instances of the *H* relation (Equation 1) corresponding to immune recognition and SHAs, respectively. *H_K_* is a total, single-valued function, whereas this study explores the extent to which *H_B_* is or is not.

We also define the *N* × *M* relation matrix **R***_ij_* according to whether the element *ϕ_i_* in Φ is associated with the element *ψ_j_* in Ψ as


$${\text{R}}_{ij}=\{\begin{array}{r} 1,\;\;\text{if yes}\;\;\;\\ 0,\;\;\text{if no}.\;\;\; \end{array}\;$$

**Figure 3:**
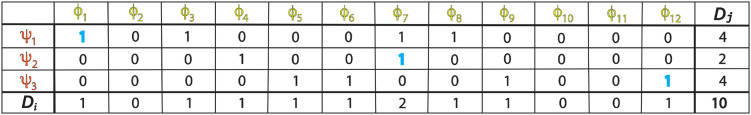
Relation matrix **R**. *ϕ_i_* and *ψ_j_*–problem and solution elements as described in **Figure 2**; ***D_i_*** and ***D_j_***– the degeneracies for problem element *i* and solution element *j*, respectively. For each possible (*ϕ_i_,ψ_j_*) pair, the corresponding matrix value indicates whether *ϕ_i_* associates with *ψ_j_* (in which case, **R***_ij_* =1), or not (**R***_ij_* =0). For example, problem element *ϕ*_1_ associates with solution element *ψ*_1_ but not with *ψ*_2_. The primary or cognate pairs are indicated with a blue”1”. The matrix elements excluding the *D_i_* =0 columns (*ϕ*_2_*,ϕ*_10_, and *ϕ*_11_) correspond to the *H* relation described in **Figure 2**. In real-world humoral immunity, many, and perhaps most, of the *D_i_*’s are 0 (e.g., solid yellow arrows in **Figure 2**; see Results Section 3.2.2). By contrast, in SHA algorithms, *D_i_* is always 1. The rows and columns of the matrix have been transposed here for illustration purposes.

See also **Figure 3**. The degeneracy, $D_{j}$, of solution element j is the number of correspondences or “yeses” across all problem elements^4^: $D_{j}$ = ${\text{R}}_{*,j}=\sum_{i=1}^{M}{\text{R}}_{ij}$, and the average degeneracy^5^ across all solution elements is $\langle D_{j}\rangle =\sum_{j=1}^{N}D_{j}/N$. See **Table 1** for a list of the variables used in this work and their definitions. Similarly, the degeneracy of problem element i is $D_{i}={\text{R}}_{i,*}=\sum_{j=1}^{N}{\text{R}}_{ij}$, and the average degeneracy across all problem elements is $\langle D_{i}\rangle =\sum_{i=1}^{M}D_{i}/M$. In immunity, $D_{j}$ is the binding degeneracy of antibody j across all epitopes, and $D_{i}$ that of epitope i across all antibodies in the repertoire. Since double sums over all ${\text{R}}_{ij}$ in the system can be carried out in either order without changing the result, we know that $\sum_{i=1}^{M}D_{i}=\sum_{j=1}^{N}D_{j}$, and hence $M\langle D_{i}\rangle =N\langle D_{j}\rangle ,\;$which is the relation size, or the sum of all the “1”s in ${\text{R}}_{ij}$. In immunity, this is the total number of possible epitope-Ab pairs involving an individual immune repertoire. Then, $\langle D_{j}\rangle =\langle D_{i}\rangle M/N$.

**Table 1:** The main variables used in this work.

Component or variable type	Problem space variable	Solution space variable
name of space	Φ	Ψ
element types	epitopes, digital files	antibodies, hash values
cardinality of the space	*M*	*N*
degeneracy of an element	*D_i_*	*D_j_*
average degeneracy of elements	⟨*D_i_*⟩	⟨*D_j_*⟩
normalized degeneracy of an element	*R_i_*	*R_j_*
collision/anticollision probability for an individual element	*P_i_*	*P_j_*
average collision/anticollision probability across all elements	⟨*P_i_*⟩	⟨*P_j_*⟩
systemic collision/anticollision probability	*P_a_*	*P_c_*
operational specificity (OpS), element	*S_i_*	*S_j_*
average element OpS across elements	⟨*S_i_*⟩	⟨*S_j_*⟩
systemic OpS	*S_a_*	*S_c_*
distribution coefficient	*K_a_*	*K_c_*
distribution coefficient, high mean	*K_a_*†	*K_c_*†
average multiplicity of *H* relation	$\bar{mult}$(*H*)	
coverage fraction of *H* relation	*f*_H_ = |Φ_H_|*/*|Φ|	
number of:		
interactions per antigen	*m*	
tested solution elements	*n*	
epitopes per antigen	*ϵ*	
tested antigens	*A*	
antigens cross-reacting with Ab	*W*	

The probability, $P_{0j}$, that a randomly chosen problem element will be associated with solution element j is $P_{0j}=\sum_{i=1}^{M}{\text{R}}_{ij}/M=D_{j}/M.$ In immunity, the probability that a randomly chosen epitope will bind to (i.e., be complementary to) antibody j is the degeneracy of that Ab as a fraction of the number of possible epitopes. The normalized degeneracy of each solution element can be given as the degeneracy relative to the mean, $R_{j}=D_{j}/\langle D_{j}\rangle$, so that $P_{0j}=R_{j}\langle D_{j}\rangle /M=R_{j}\langle D_{i}\rangle /N$. Similarly, the normalized degeneracies of the problem elements are $R_{i}=D_{i}/\langle D_{i}\rangle$, and the probability that a randomly chosen Ab will bind to epitope i is $P_{0i}=\sum_{j=1}^{N}{\text{R}}_{ij}/N=D_{i}/N=R_{i}\langle D_{i}\rangle /N=R_{i}\langle D_{j}\rangle /M.$

#### 2.2.2 Operational specificity

If an antigen contains *ϵ_i_* epitopes, *E_i_* = {*i*_1_*,i*_2_*,…i_ϵ_i__*}, then the number of Ab interactions it will have is $m_{i}=\sum_{k=1}^{\epsilon_{i}}1_{\left\{D_{i_{k}}=1\right\}}$. Assuming $D_{i}$ is usually 0 or 1 for most epitopes (see Section 4.2.2), then $\langle D_{i}\rangle <1$ and, very approximately, $m_{i}\;\approx \langle D_{i}\rangle \epsilon_{i}$, Over all antigens, the average number of Ab interactions per antigen, 〈m〉, will more closely approximate $\langle m\rangle \approx \langle D_{i}\rangle \langle \epsilon \rangle$. Hence, for $\langle D_{i}\rangle <1$, a fair approximation is $\langle D_{i}\rangle \approx \langle m\rangle /\langle \epsilon \rangle$.

To define operational specificity, or OpS, we first establish the idea of primary or cognate pairs, which are problem element-solution element pairs that we define to be elements of a “special” or primary subset of the overall relation and that are uniquely paired. By “uniquely paired”, we mean they form a partial bijection or a bijective subset of the overall relation. Namely, they are a subset, *H′*, of *H:*


$$H^{\prime }=\{\left(\phi_{g(j)},\psi_{j}\right)|j\in \left\{1,2,3,\ldots N\right\}\},$$

where *g*: Ψ → Φ*_cog_* is a bijection (unique pairing) and Φ*_cog_* is the subset of Φ for which each element is cognate to a corresponding element in Ψ. This assumes that *M* ≥ *N*. See **Figure 2**. The set of pairs of tested epitopes and their cognate antibodies is a cognate subset, as is the set of pairs of digital messages to be secured and their corresponding hash values.

As shown in **Table 2**, there are three possible relationships between a cognate ordered pair $\left(\phi_{i_{1}},\;\psi_{j_{1}}\right)\;$and any other ordered pair $(\phi_{i_{2}},$ $\psi_{j_{2}})$. For simplicity, the table assumes that g(j)=j. That is, the indices of cognate problem and solution elements are equal. 1) If $i_{1}\neq i_{2},\;j_{1}=j_{2}$, the pairs share only the same solution element, and we call the relationship a *collision* or *cross-reaction* (solid, black arrows in **Figure 2**); 2) If $i_{1}=i_{2},\;j_{1}\neq j_{2}$, the pairs share only the problem element, and the relationship is an anticollision (dashed, black arrow in **Figure 2**; see also Section 3.5). Finally, 3) If $i_{1}\neq i_{2},\;j_{1}\neq j_{2}$, the pairs share neither element and participate in a non-collision. Throughout this work, the term “collision” will be assumed to include the idea of antibody cross-reaction with non-cognate epitopes, and “specificity” will refer to collision specificity, rather than anticollision specificity, unless otherwise stated. Notably, there are no anticollisions in SHA algorithms; the present study explores how close humoral immunity comes to this, if at all.

**Table 2:** Examples of the three types of relationships between a cognate ordered pair and other ordered pairs (assuming i = j for cognate ordered pairs).

Relationship	Cognate ordered pair	Other ordered pair	Example
collision	(*ϕ*_1_*, ψ*_1_)	(*ϕ*_2_*, ψ*_1_) (non-cognate pair)	an antibody that cross-reacts with a non-cognate epitope
anticollision	(*ϕ*_1_*, ψ*_1_)	(*ϕ*_1_*, ψ*_2_) (non-cognate pair)	an epitope that cross-reacts with a non-cognate antibody
non-collision	(*ϕ*_1_*, ψ*_1_)	(*ϕ*_2_*, ψ*_2_) (cognate pair) or (*ϕ*_2_*, ψ*_3_) (non-cognate pair)	two antibody-antigen pairs which are distinct in both elements

**Figure 4:**
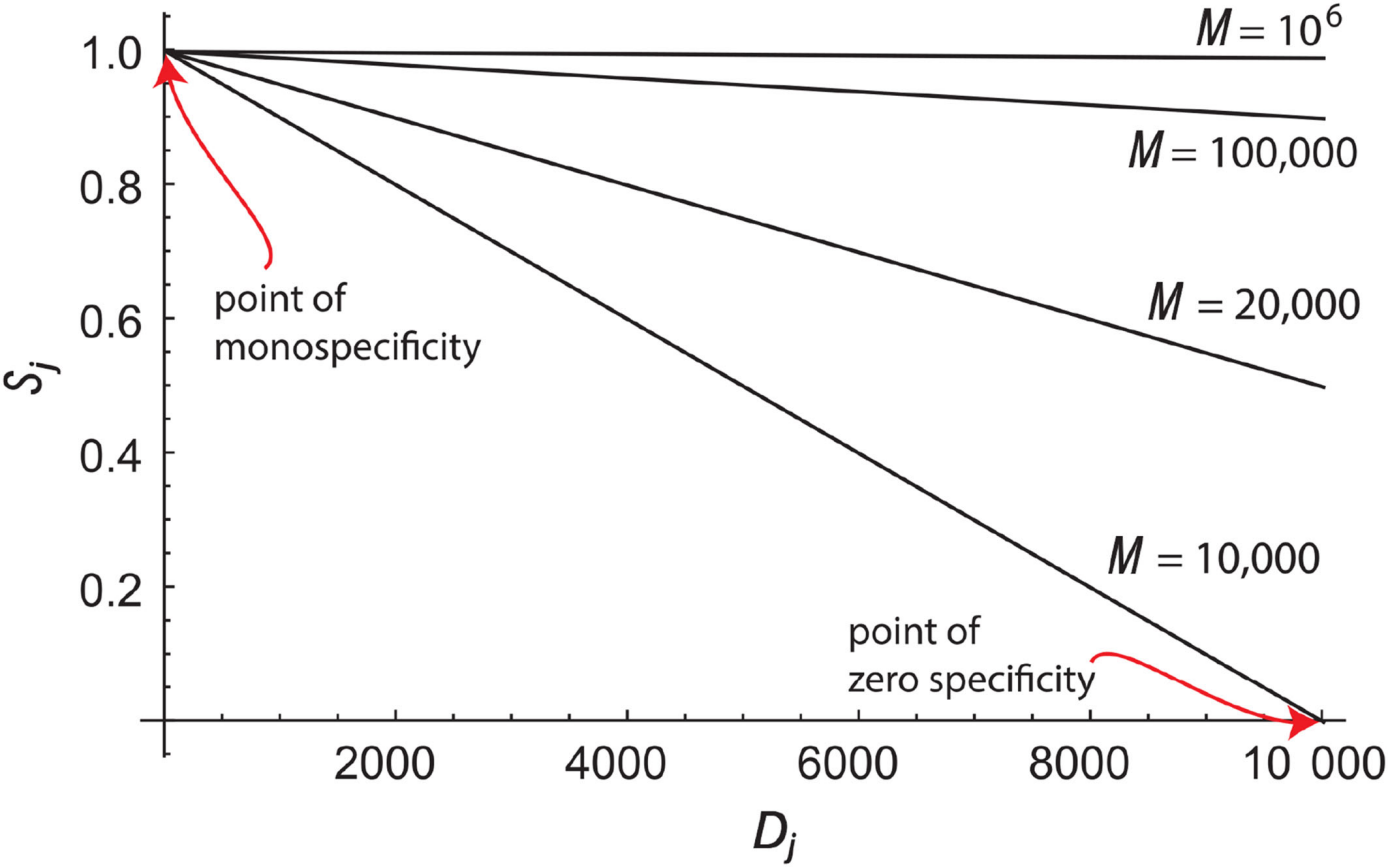
Dependence of specificity (OpS) on degeneracy. The operational specificity, *S_j_*, of a solution element (e.g., an antibody or hash value) for its cognate problem element (e.g., epitope or digital file), relative to a randomly selected problem element, is plotted on the vertical axis as a function of its degeneracy, *D_j_*. The various lines represent different sizes, *M*, for the problem space, which determine the line’s slope. The point (*D_j_,S_j_*) = (1,1), labeled the “point of monospecificity,” is the only point where the solution element is absolutely specific for its cognate problem element. It is also where the specificity is independent of the size of problem space. When *D_j_* = *M*, (e.g., *D_j_* =10,000 for *M* =10,000), the specificity is zero.

The operational specificity of an element, *S*, measures how unlikely it is for the element to participate in a collision or cross-reaction. For individual elements or their averages, *S* = 1 − *P*, where *P* is the probability of a collision^6^. If *P_j_* = 0 and *S_j_* = 1, then no non-cognate problem elements point to solution element *j*, and it has perfect specificity for its cognate problem element. In immunity, this would mean an antibody is truly monospecific. In cryptography, no alternative files would hash to primary or originating message digest *j*. Conversely, *S_j_* = 0 implies that all problem elements collide: all non-cognate epitopes cross-react with antibody *j* and all alternative files hash to message digest *j*. This is illustrated in **Figure 4**.

Collision probabilities and OpS can be considered in the context of individual antibodies, *P_j_, S_j_*, or system averages, ⟨*P_j_*⟩, ⟨*S_j_*⟩. In addition, the *systemic probability of a collision*, *P_c_*, is the probability of a cross-reaction between a solution element and one non-cognate problem element anywhere across the entire solution space, and the *systemic OpS*, *S_c_*, is the corresponding specificity.

For individual solution elements and $\;D_{j}\gg 1$, $P_{j}\approx \frac{D_{j}}{M}=P_{0j}$ and $S_{j}\approx 1-\frac{D_{j}}{M}$. Hence, as depicted in **Figure 4**, the specificity is a function of the degeneracy and the size of the problem space. The latter expression is similar in form, though not exactly the same, as the measure called specificity used in binary medical tests (60).^7^ Similarly, For $D_{j}\gg 1$, averages across the system are

(2) $$\langle P_{j}\rangle \approx \frac{\langle D_{j}\rangle }{M}=\frac{\langle D_{i}\rangle }{N}$$

and $\langle S_{j}\rangle$ is 1 minus those quantities.

For large problem/solution spaces, systemic OpS is generally *S_c_* ≈ *e*^−^*^Pc^*, which reduces to *S* ≈ 1 − *P_c_* for *P_c_* ≪ 1. The forms for *P_c_* and *S_c_* in terms of other system variables, as well as all derivations, are provided in the Appendix (Section 6.2) and **Supplementary Material 3**.

#### 2.2.3 Phenomenological simulations related to systemic OpS

In this set of calculations, *N* antibodies in the system were assigned degeneracies (*D_j_*’s) conforming to a positive-valued Gaussian distribution. Then, *D_j_* epitopes were randomly associated with each antibody, *j*, one Ab per epitope (*D_i_* =1). Pairs of epitopes were then selected at random–the first representing the cognate epitope in an antibody-epitope pair. If the second happened to bind the same antibody as the first, then the epitope pairing was counted as an Ab cross-reaction. This was repeated for the entire set of epitopes, so that there were a maximum of 100,000 ×99,999/2 ≈ 5 x 10^9^ epitope pairs per trial. The probability of cross-reaction was calculated as the number of positive cross-reactions divided by the total number of epitope pairs, and this was compared to the theoretical result. A number of trials were carried out, varying the spread of the degeneracies (*σ* of the Gaussian distribution). The actual number of epitope pairs per trial varied between 2 and 5 billion, because of the effect of truncating the Gaussian (at *D_j_*=0), which varied with the spread parameter.

### 2.3 Antibody-epitope interaction probability model

#### 2.3.1 AEIP model form

The above models do not take into account sampling of subsets of antibodies from larger pools, as occurs in polyclonal immune responses to an antigen. To guarantee the generation of accurate statistics for multi-epitope, multi-antibody interactions involving such sampling across all size scales, the antibody-epitope interaction probability (AEIP) model was developed. This model generates the probability, *P*(*ϵ, m, n, N*), of an antigen having *ϵ* epitopes that will participate in *m* interactions with a set of *n* distinct antibodies or B-cell clones selected from a larger pool of *N* clones in the immune repertoire. The total number of expected complementary interactions, or “matches,” ⟨*W*⟩, given *A* tested antigens, is then simply ⟨*W*⟩ = *AP.* The assumptions are that 1) *H_B_* is total (no unassigned epitopes), 2) *H_B_* is random; 3) the antigens are each assigned random epitopes, 4) duplicate epitope-antibody matches for a given antigen are not allowed (no combinatoric replacement), and 5) the antigenic binding spaces of the Abs are of the same size (all *D_j_* are equal). This last condition is why the Ab degeneracies do not appear explicitly in the model. Conditions 2, 3, and 5 imply that the antigenic space is apportioned more-or-less evenly among the *N* antibodies.

The probability is the product of four terms:

(3) $$P\left(\epsilon ,m,n,N\right),=S_{\epsilon }C_{n}T_{1}T_{2}$$

where


$$S_{\epsilon }=\frac{\epsilon !}{\left(\epsilon -m\right)!},\;C_{n}=\frac{n!}{\left(n-m\right)!m!},\;T_{1}=\frac{(N-n)!}{\left(N-n-\epsilon +m\right)!},\;T_{2}=\frac{(N-\epsilon )!}{N!},$$

provided that the arguments of the factorials are all greater than zero–i.e., *N* ≥ {*ϵ,n*} ≥ *m*, and *N* ≥ *n* + *ϵ* − *m*. This expression is exact, in the sense that statistical results will converge to it over a large number of trials.

In the special case of *n* =1 (a single selected or tested Ab), the number of cross-reactive matches, *m*, can be either 0 or 1, and the probability reduces to


$$P(\epsilon ,m,1,N)=\{\begin{array}{l} 1-\epsilon /N\text{ if }m=0,\\ \epsilon /N\text{ if }m=1, \end{array}$$

and ⟨*W*⟩ = *Aϵ/N* for a single match. Since ⟨*D_i_*⟩ = 1 in the AEIP model, from Equation 2 the average probability of collision for an individual Ab is ⟨*P_j_*⟩ ≈ 1*/N* and ⟨*W*⟩ = *Aϵ*⟨*P_j_*⟩, as in the example given in Results Section 3.7.

As discussed in the Appendix (Section 6.3), for arbitrary *n* ≥ *ϵ* ≥ *m >* 0 and *N* ≫ {*n,ϵ,m*}, the probability simplifies to *P*(*ϵ,m,n,N*) ≈ *S_ϵ_C_n_/N^m^*.

Various other approximations to the exact model are derived and other details are also provided in the Appendix (Section 6.3).

#### 2.3.2 Phenomenological simulations related to the AEIP model

**Figure 5:**
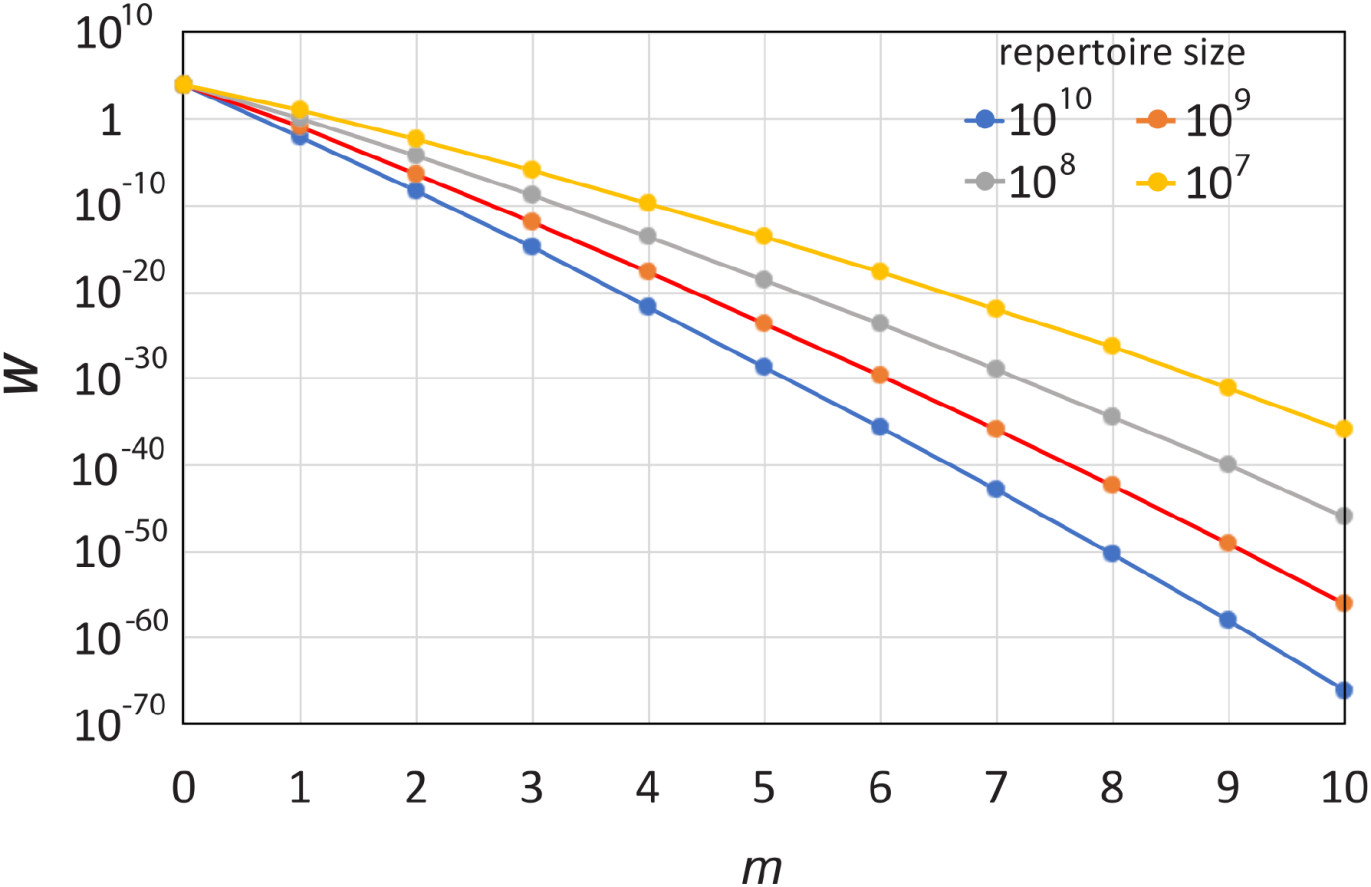
Log plot of the probability of interaction between antibodies raised in a polyclonal response to a non-self antigen and the set of all self-antigens in the human body, according to the AEIP model. *W*–the average number of self-antigens, out of 10000, that will likely interact with any of 10 selected antibodies, assuming 1000 epitopes per Ag, for various sizes of antibody repertoires (base-10 log plot). *m*–the number of cross-reactions per antigen. It is unlikely for even one self-antigen to find two Ab matches, and the probabilities decrease exponentially from there with the number of matches.

Several sets of trial calculations, or phenomenological simulations, were carried out to quantify the probabilities of interaction between sets of selected antibodies and arbitrary antigens, and the results were compared to the theoretical estimates from the AEIP model. For each calculation, a set of *n* antibodies was randomly selected out of a larger pool of *N* Abs, which also correspond to the *N* partitions into which epitope space was subdivided. Then, *ϵ* epitopes were randomly selected from those partitions and assigned to a test antigen and checked for complementarity with the *n* selected Abs. The number of non-redundant matches was then tabulated for each of *A* test antigens. The results were compared with the theoretical results, using the exact formulation (log form of Equation 3) and either of four approximations for the probabilities, which are described in the Appendix (Section 6.3). For most trials, *ϵ* = 5 was used, because that is a typical number of immunodominant epitopes involved in an immune response (61, 62) and it also allows for smaller repertoire sizes to be explored, given the constraint *N > ϵ*. In one set of trials (see **Figure 5**), *ϵ* =1000, which is a high-end estimate of the number of recognizable epitopes on an antigen (**Supplementary Material 4**).

## 3 Results

### 3.1 Size of the problem domains, Φ

#### 3.1.1 The size of electronic file space

The size of digital file space grows exponentially with file size. The contents of a 4000-bit input file can be arranged in 2^4000^ or approximately 10^1204^ ways, and hence the size of the file space, *M* = 10^1024^. For comparison, the number of particles in the known universe is very approximately 10^80^.

#### 3.1.2 The size of peptide/protein epitope space

The number of possible epitopes that the humoral immune system could be tasked with recognizing also grows roughly exponentially with molecular or fragment size. As described in Methods, the PECS model gives a lower-bound estimate for the size of epitope space as *M_prot_* = (*N_r_d*)*^q^*, where *N_r_* is the number of residue types, *q* is the number of (*x,y*) positional “slots” for the amino acids across the binding interface and *d* is the number of possible *z*-positions, which are the depths of the *α*-carbons relative to the binding interface.

This is illustrated in **Figure 1**. As to an estimate of *q*, multiple studies have shown that the average protein or peptide epitope–i.e., the set of amino acids interacting at the antibody-antigen interface–consists of about 15–25 residues (58, 59, 63), and many epitope interfaces contain 30 amino acids or more. Because a reasonable lower bound is sought here, we choose 15 as the maximal number of amino acids and, in addition, we limit the number of positional slots to the number of amino acids, so that *q* =15. Since any of 20 possible amino acid types can occur at each of those (*x,y*) slots, *N_r_* =20. To estimate *d*, we partition the depth of residues relative to the hypothetical interfacial plane into a number of regions, as shown in the figure. The great majority of epitope amino acids are centered at a Chakravarty depth (distance of an atom from the nearest surface water molecule) of 8Å or less, and most are between 3.5 Å and 6 Å (64). Hence, we can reasonably discretize the problem by allowing the *α*-carbon of an amino acid to occupy any one of three depths relative to the epitope surface, each separated by roughly 2.0-2.5Å. This separation is large enough to capture typical local fluctuations, as measured, for example, by RMS deviations of *α*-carbons in MD simulations of stable structures (65, 66), or between homologous *α*-carbons in conserved regions of different proteins (67).

The depth of the epitope surface, itself, can also vary relative to the interfacial plane. Since, again, we are erring on the side of undercounting possible configurations, we suppose only three different possible depths for the surface at each amino acid position and assume the separation to be roughly equal to that between the possible depths of the amino acids relative to the surface. Hence, each residue can be at any of *d* =5 depths relative to the plane (any of 3 possible positions relative to the surface, with two possible shifts of the surface). Further, since epitopes can be discontinuous, the model assumes the amino acid positions are all independent of each other. Hence, the overall estimate arising from the model is *M_prot_* ≈ (20 · 5)^15^, or 10^30^. For multiple reasons cited above and in the Appendix (Section 6.1), this is likely a very conservative lower-bound estimate for the number of possible protein epitopes that the adaptive immune response must be capable of recognizing/binding.

#### 3.1.3 The size of hapten space

In addition to proteins and peptides, the immune system recognizes any number of molecular types, including sugars, lipids, carbohydrates, drugs and small molecules. These molecules can function as immunogens, provided they are coupled with carrier proteins. It is estimated that there are about 10^63^ possible small organic compounds of molecular weight 500 Da or less that are stable in water at room temperature, if only C, H, O, N, P, S and halide atom types are included (68). Restricting our analysis to molecules of this size and assuming only one conformation per molecule, we can set 10^63^ as the lower bound for the number of possible haptens that the immune system is tasked with recognizing. Further, assuming that carrier proteins contribute up to 10 amino acids to the combined hapten/protein epitope and using the PECS model described above for the chemical and conformational diversity of the amino acids, the total number of possible, distinct structures comprised of hapten and protein is *M* ≈ 10^63^ × (20 · 5)^10^or about 10^83^. This likely represents a very conservative, lower-bound estimate of the number of possible molecular structures to which the immune system could be challenged to respond, because 1) larger haptens (e.g., digoxin at a M.W. of 781 Da) (69), haptens containing different atom types (70, 71), and larger protein epitopes (72) are known to exist, and 2) the estimate does not take into account the conformational diversity of the haptens. In addition, *M* or |Φ| is likely to be significantly larger than the number of structures, because humoral immunity generally recognizes multiple epitopes on each hapten-carrier conjugate. Put another way, although antigenic totality suggests *M* is at least as large as the number of hapten/carrier protein structures, it could be larger (see also Glossary, **Supplementary Material 2**).

### 3.2 Size of the repertoires (Ψ) and the degeneracies of *ψ_j_*

As described in Methods (Section 2.2), the average degeneracy of solution elements (e.g., Abs) is ⟨*D_j_*⟩ = ⟨*D_i_*⟩ *M/N*, where *M* = |Φ| and *N* = |Ψ| are the problem and solution set sizes, and ⟨*D_i_*⟩ is the average degeneracy of the problem elements (e.g., epitopes).^8^ When ⟨*D_i_*⟩< 1, it can be considered a measure of the coverage fraction of the *H* relation, *f*_H_ = |Φ_H_|*/*|Φ| –that is, the “completeness” of the binding repertoire. On the other hand, when ⟨*D_i_*⟩ *>* 1, it is a measure of the “multivalued-ness” or multiplicity of *H*–e.g., the binding space overlap of the antibodies.

#### 3.2.1 A hash function’s repertoire

In the cryptographic case, ⟨*D_i_*⟩ is the average degeneracy of all files or messages, and because hash functions (*H_K_*) behave as total mathematical functions–i.e., each digital file maps to one and only one hash value– *D_i_* = ⟨*D_i_*⟩ = 1, and the average degeneracy of the hash values reduces to ⟨*D_j_*⟩ = *M/N.* For our example case involving SHA-256, *M* ≈ 10^1204^, the size of the solution domain is *N* = 2^256^ ≈ 10^77^, and ⟨*D_j_*⟩≈ 10^1204^*/*10^77^ = 10^1127^. Hence, the *H_K_* relation (here, SHA-256) is highly many-to-one or non-injective. In this absolute sense, hash values are not at all specific to a given file.

#### 3.2.2 The antibody repertoire

Analogously to the cryptographic case, if *M* is the number of (distinct) epitopes, *N* the number of (distinct) antibodies or cellular receptors, and ⟨*D_i_*⟩ the average degeneracy of epitopes with respect to an individual’s immune repertoire, then the average Ab degeneracy in the system is ⟨*D_j_*⟩ = ⟨*D_i_*⟩ *M/N*. The estimate for *M* was given above. Now, we estimate *N* and ⟨*D_i_*⟩.

There are ≈ 10^11^ to 10^12^ T and B cells in the human body (73, 74) and because there tend to be multiple copies of each cellular clone, the number of chemically distinct antibodies/immune receptors in an individual–i.e., the size an individual’s immune repertoire, *N*–is thought to be^9^ in the range of 10^7^ to 10^10^ (75–78).

As described in Methods (Section 2.2), a fair estimate of ⟨*D_i_*⟩ is ≈⟨*m*⟩*/*⟨*ϵ*⟩, where *m* is the number of antibody interactions per antigen and *ϵ* is the number of epitopes per antigen. It is known that different antibodies can bind similar epitopes (79–84), but in this work, an epitope is defined such that similar, but distinct chemical compounds are counted as different epitopes. We know that *D_i_* is often< 1, since individual immune responses tend not to produce antibodies against all epitopes on an antigen (85–89). As discussed in Section 3.7 below and in **Supplementary Material 4**, a generous estimate for ⟨*ϵ*⟩ is 1000, and individual immune responses typically generate antibodies to a few tens of epitopes (⟨*m*⟩), so a reasonable lower bound for ⟨*D_i_*⟩ is ≈ 10*/*1000=1*/*100. For simplicity, throughout this work ⟨*D_i_*⟩ = 1 is often used as a first approximation for the immunologic case.

Combining estimates for *M, N*, and ⟨*D_i_*⟩, a conservative lower bound estimate for ⟨*D_j_*⟩ is a range of ≈ (1*/*100) × 10^83^*/*10^10^ = 10^71^ to 1 × 10^83^*/*10^7^ = 10^76^. This is a very low-end estimate of the number of epitopes, as defined here, that each Ab species, on average, is tasked with being able to bind. The number of protein/peptide epitopes is likely to be at least ⟨*D_j,prot_*⟩ ≈ (1*/*100) × 10^30^*/*10^10^ to 1 × 10^30^*/*10^7^ = 10^18^ to 10^23^. Hence, the *H_B_* relation is highly many-to-one as well, at least on average.

These are fairly robust results. Using a much more restrictive approximation of the size of chemical space (90, 91) for the size of the hapten domain changes the conclusions quantitatively but not qualitatively.

### 3.3 Operational specificity

We define operational specificity (OpS), *S*, as the unlikelihood of an element pairing with a non-cognate partner (See Methods, Section 2.2.2) It can be considered in at least three contexts: 1) that of averages over all solution elements, 2) that of individual solution elements and 3) that of the system as a whole. We will consider each in turn, here. We discuss anticollision probabilities and epitope OpS, with the corresponding results, in Section 3.5 and **Supplementary Material 3.2**.

#### 3.3.1 Average OpS over all solution elements

**Figure 6:**
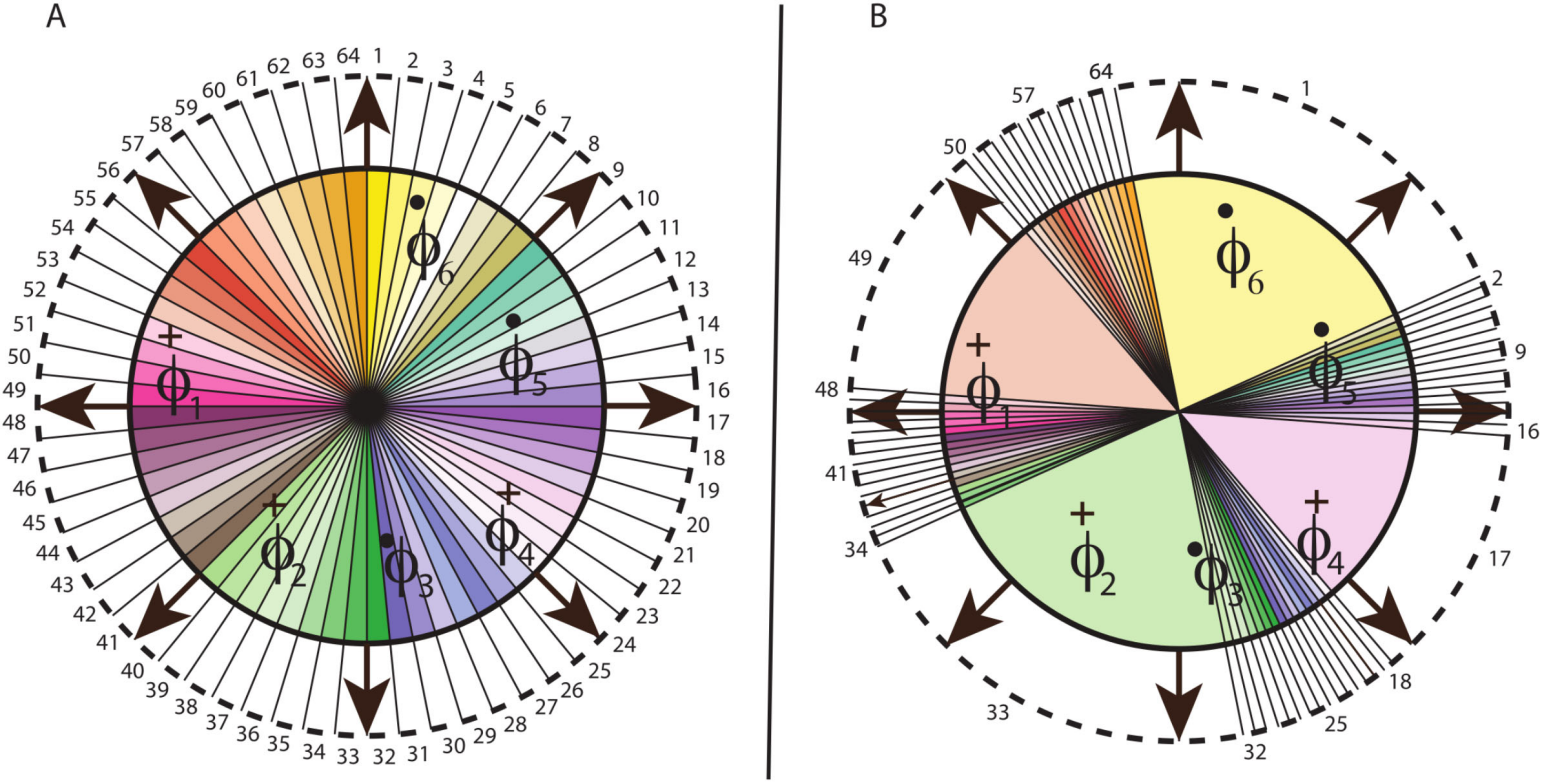
Binning of chemical (or message) space. For both panels, the inner, colored circular area represents the set of possible epitopes; each of the 64 circular sectors (out to the dashed circular boundary) is the slice of chemical space to which each distinct Ab is complementary, assuming no overlap and complete coverage; *ϕ***_1_**through *ϕ***_6_**–different epitopes; crosses (+)–epitopes cognate to their respective antibodies; dots (•)–random, non-cognate epitopes. In **(A)** the probability that a randomly selected (•) epitope would be in the same bin as a cognate (+) epitope is 1/64, because the chemical space is divided equally. In **(B)** four antibodies dominate the space, so that the odds of such a cross-reaction are much higher. In this way, the probability of a cross-reaction or collision increase with the variance in the degeneracies. In cryptology, the circle represents the set of all possible digital messages that a hash function could receive as input; each slice represents a subset of messages that result in a particular digest or hash value. For SHA-256, there would be ≈10^77^ slices. In humoral immunity, there are 10 million slices, or more. An expansion of the set of possible epitopes or digital files, depicted here as an enlargement of the colored circular area to the dashed outer circle, does not change the probability of a cross-reaction or collision, provided the new *ϕ* are randomly distributed across the solution space.

Since the human immune repertoire contains 10^7^ to 10^10^ distinct Ab variable region species (*N*), conservative, lower bound estimates of $\langle P_{j}\rangle =\langle D_{i}\rangle /N$ are $\approx 10^{-10}$ to $10^{-7}$ for $\langle D_{i}\rangle =1$, and $10^{-12}$ to $10^{-9}$ for $\langle D_{i}\rangle =1/100$. The corresponding estimates for $\langle S_{j}\rangle$ are a range of $1-10^{-7}$ to $1-10^{-10}$ and $1-10^{-9}$ to $1-10^{-12}$, respectively. It is in this sense that antibodies are, at least on average, highly specific. As depicted in **Figure 6**, cross-reactivity is improbable for each randomly selected antibody-epitope, on average, though not nearly as improbable as a random collision (second preimage) in a cryptographic algorithm such as SHA-256. In the case of SHA-256, ⟨*S_j_*⟩ is about 1-10^-77^. The statistical comparison between the two systems is summarized in **Table 3**.

**Table 3:** Basic statistical comparison between the SHA-256 model system used in this study (SHA-256 System column) and the B cell receptor/antibody immune recognition system (Humoral Immunity column).

Component or variable	SHA-256 system	Humoral immunity
problem element	digital file	epitope
solution element	hash value	antibody
M	10^1204(^*^a^*^)^	10^83^
$M_{prot}$		10^30^
N	10^77^	10^7^ to 10^10^
$\langle D_{j}\rangle$	10^1127(^*^a^*^)^	10^73^ to 10^76^
$\langle P_{j}\rangle$	10^-77^	10^–12^ to 10^-7^
$\langle S_{j}\rangle$	1-10^-77^	1-10^–7^ to 1-10^-12^
$P_{c}$	10^-77^	10^–12^ to 10^-7^
$S_{c}$	1-10^-77^	1-10^–7^ to 1-10^-12^
$n_{c}\;$	10^22^	10^2^
$P_{j}|$ $n_{c}$	10^-55^	10^–10^ to 10^-5^
$\langle D_{i}\rangle$	1	0.01 to 1
$\langle P_{i}\rangle$	0	10^–14^ to 10^-8(^*^b^*^)^
$\langle S_{i}\rangle$	1	1-10^–8^ to 1-10^-14(^*^b^*^)^
$P_{a}$	0	10^49^ to 10^59(^*^b^*^)^
$S_{a}$	1	≈0
$\bar{\text{mult}}\left(H\right)$	1	1.005 to 1.58^(^*^b^*^)^
$f_{\text{H}}$	1	0.01 to 0.63^(^*^b^*^)^

M_prot_–estimated number of distinct protein/peptide epitopes. n_c_ –number of solution elements generated in response to a typical challenge. For the SHA, the example used is the number of hash value calculations that can be performed on 100 Bitcoin mining machines over 2 weeks as of ≈ 2023. For the immune system, it is the number of distinct epitopes eliciting cognate Ab production in a typical viral infection. **P_j_| n_c_**– The probability that a typical challenge will result in a collision with a fixed hash value target (and corresponding file) or a cross-reactive match between the set of elicited antibodies and a given (e.g., self) epitope. The rest of the row headings are as per **Table 1**. Although the magnitudes of the results are different in the two systems, the mathematical structure is very similar, diverging only for P_a_ and S_a_, the systemic probability of anticollision and the corresponding OpS (see Results Section 3.5, Equation 5, and Discussion 4.2).. (a) Assuming a message size of 4000 bits (250 16-bit words). (b) Assuming a Poisson distribution for epitope degeneracies (D_i_).

#### 3.3.2 Operational specificity of individual solution elements

As derived in the Appendix, Section 6.2, for high solution element degeneracies, ⟨*D_j_*⟩ ≫ 1, the collision probability for an individual solution element (antibody or hash value), *j*, is *P_j_* ≈ *R_j_*⟨*D_i_*⟩*/N*, where *R_j_* is the normalized degeneracy of solution element *j.* This holds provided there are no prior correlations between problem and solution elements. In the case of SHA functions in current use, the distribution of the *M* files among the *N* − 1 non-cognate hash values is close to random and uniform (92–94). Although the output of these functions is exactly reproducible for each unique input, it varies chaotically with small changes in the input, in what is known as the *avalanche effect* (95), resulting in a pseudo-random distribution. And since the solution elements (hash values) are all of the same size, this pseudo-random mapping also ensures that each member of the solution set has very nearly the same number of files mapping to it (preimage cardinality). Thus, the probability distribution of *R_j_* is spiked, with all *R_j_* ≈ 1. Further, since ⟨*D_i_*⟩ = 1, it is clear that *P_j_* = ⟨*D_i_*⟩*/N* ≈ 1*/N*, for each hash value, *j*. This is analogous to randomly assigning *M* possible problem elements into *N* equally sized bins, as depicted in **Figure 6**. The OpS for each hash value is then *S_j_* ≈ 1 − 1*/N* = ⟨*S_j_*⟩, which for the example case is, again, ≈1-10^-77^.

The situation in immunity is analogous. Absent prior exposure, the distribution of epitopes across the repertoire of *N* − 1 non-cognate antibodies is likely very close to random, because the recombination of the coding segments for antibodies is known to be largely random (16, 17). In addition, small changes in structure tend to have disproportionate effects in antibody-antigen affinity (96–101), in what could be called the immunological version of the avalanche effect. Hence, in the general case, two epitopes with structures that vary more than slightly are no more likely to bind the same antibody than by chance.

As to the size distribution of the binding spaces of individual antibodies, there is a paucity of data, but the distribution of CDR3 lengths, which has been considered a proxy for binding site diversity, is reported to be roughly a truncated Gaussian (76, 102, 103). In any symmetric distribution of positive-valued data, the largest data point value cannot exceed twice the mean (because *x*_max_ = 2 × mean − *x*_min_, and *x*_min_*>* 0). Hence, a size distribution that is approximately a truncated Gaussian, or otherwise symmetric, implies a maximal normalized degeneracy for solution elements of *R_j,max_* ≈ 2, and a maximal cross-reaction probability of *P_j,max_* ≈ 2⟨*D_i_*⟩*/N* = 2⟨*P_j_*⟩. The minimal OpS of an antibody taken from a symmetric distribution of Ab degeneracies is then *S_j,min_* ≈ 1−2⟨*D_i_*⟩*/N*. Assuming, again, that ⟨*D_i_*⟩ = 1, this means *S_j,min_* =1 - 2 × 10^–7^ to 1 - 2 × 10^-10^, which is the same order of magnitude as the average OpS across all antibodies (1-10^–7^ to 1-10^-10^). Thus, truncated Gaussian or other symmetric binding space distributions do not, in general, lead to order-of-magnitude drops in individual Ab operational specificities, relative to the mean.

At the other extreme, the bounds for the maximal OpS (*S_j,max_*) and minimal cross-reactivity (*P_j,min_*) for individual antibodies are less clear. While affinity differences between different antibodies have been quantified–e.g., affinity maturation may confer an increase in binding affinity of one or two orders of magnitude (86, 104, 105)–differences in binding space sizes or specificities have not.

#### 3.3.3 Systemic OpS

**Figure 7:**
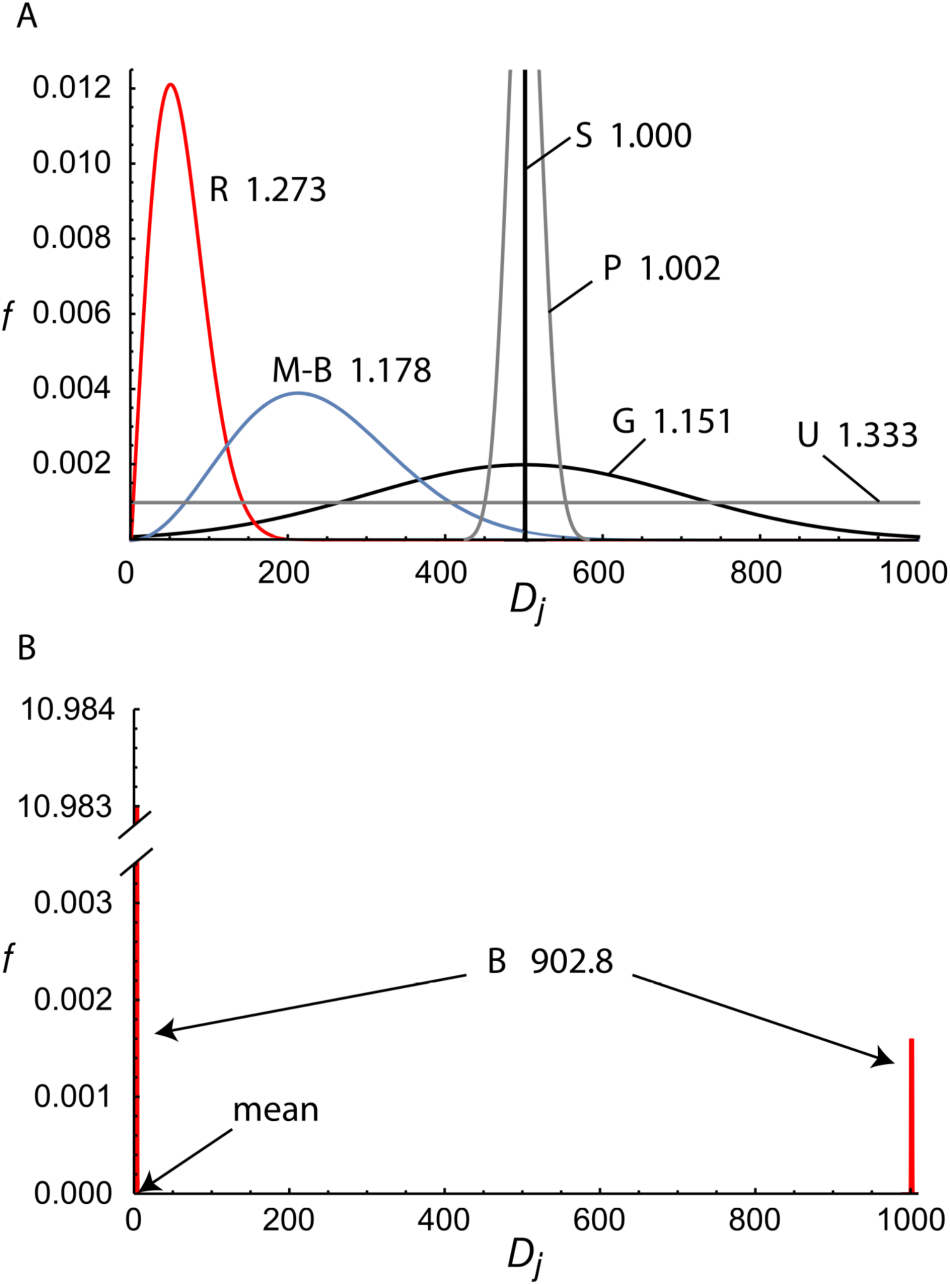
Unimodal and bimodal distributions for antibody or hash value degeneracies. Panel **(A)** shows five different unimodal distributions for solution element degeneracies, normalized to the interval *D_j_* ∈ [0,1000], and their associated (high-mean) distribution coefficients, *K_c_*^†^. They are: R–a Rayleigh distribution (red curve) with *σ* =50; M-B–a Maxwell-Boltzmann distribution (blue curve) with *σ* =150; G–a Gaussian (black curve) with *σ* =200 and *µ* = ⟨*D_j_*⟩ = 500; S–a singular distribution (black spike) at *D_j_* =500, P–a Poisson distribution (grey curve) with *λ* = ⟨*D_j_*⟩ = 500,; and U–a uniform distribution (grey line). The singular distribution minimizes the variance and, hence, the distribution coefficient, and it therefore maximizes the system specificity. However, the distribution coefficients of the other unimodal curves do not differ from that optimal case by more than a factor of 1.333 in these examples, despite their varying forms. By contrast, Panel **(B)** shows a skewed and widely split bimodal distribution (red spikes, “B”) in which a small number of elements (100) account for most (95.0%) of the system’s degeneracy and the vast majority (100,000) account for very little, resulting in a large variance and *K_c_*^†^ (902.8, as well as *K_c_* =901.9). This greatly diminishes the system OpS and increases the chances for cross reactivity or collision relative to the optimal case.

The systemic OpS takes into account all possible pairwise combinations of members of the problem repertoire (e.g., epitopes) with all (single) elements in the solution (Ab) repertoire. As detailed in the Appendix (Section 6.2), assuming large problem spaces (M ≫ 1) and solution element degeneracies (⟨D_j_⟩≫ 1), and assuming complementary (ϕ,ψ) pairings are uncorrelated, the systemic probability of collision, P_c_, is approximately

(4) $$P_{c}\;\approx \;{\frac{\langle D_{i}\rangle }{N}}^{2}(\text{Var}(R_{j})+1)=\frac{{\langle D_{j}\rangle }^{2}N}{M^{2}}(\text{Var}(R_{j})+1),$$

where (Var(*R_j_*) + 1) = $K_{c}^{†}$ is the high-mean distribution coefficient for solution elements. The systemic OpS is given by *S_c_* ≈ 1−*P_c_*, provided *P_c_* ≪ 1. As also discussed in the Appendix (Section 6.2), the *P_c_* term is minimized, and *S_c_* is maximized, when the probability distribution of *D_j_* is singular (i.e., “spiked”; see **Figure 7**), and all *R_j_* =1, so that the variance Var(*R_j_*) is essentially zero and $S_{c}\approx 1-P_{c}=1-\frac{{\langle D_{i}\rangle }^{2}}{N}$. Hence, in the case of a spiked distribution, $K_{c}^{†}$ = 1. Notably, cryptographic hash algorithms such as SHA-256 are thought to have a spiked preimage size distribution (93, 106). For our example cryptographic case, then, we can estimate *P_c_* for the hash function to be the same as ⟨*P_j_*⟩, i.e., $P_{c}\approx \frac{{\langle D_{i}\rangle }^{2}}{N}=\frac{1^{2}}{10^{77}}=$10^-77^ ≈ $\langle P_{j}\rangle$ and, similarly, $S_{c}\approx \langle S_{j}\rangle \approx$1-10^-77^.

From Equation 4, note that as *M* is increased, so long as the solution element degeneracies, *D_j_*, increase proportionately, *P_c_* remains unchanged, as Var(*R_j_*) is constant under uniform scaling of *D_j_*. Hence, similar to the case for the average antibody OpS, as long as *H_B_* is random, the systemic probability (*P_c_*) and specificity (OpS) are unchanged as the number of epitopes to which the system is exposed (*M*) is increased. See also **Figure 6** and Appendix (Section 6.2).

As mentioned above, the actual size distribution of Ab binding spaces is unclear, but there is some data to suggest that it is approximately Gaussian. Since the maximal variance of any Gaussian distribution over its positive support is the mean squared or, here, ⟨*R_j_*⟩^2^ (107–109), and since, by definition, ⟨*R_j_*⟩ = 1, it must be true that max(Var(*R_j_*)) = 1^2^ = 1 for Ab binding degeneracies conforming to Gaussian distributions. Hence, the maximal $K_{c}^{†}$ for a Gaussian distribution of Ab degeneracies is 2, and for large ⟨*D_j_*⟩ and fixed *N* and ⟨*D_i_*⟩, the maximal probability of a collision across the system is $\;P_{c,max}=\frac{2{\langle D_{i}\rangle }^{2}}{N}\;,\;$or twice the optimal value, and the minimal OpS, $s_{c,min}=1-\frac{2{\langle D_{i}\rangle }^{2}}{N}$. Further, what is conventionally considered a Gaussian distribution of positive data generally has a location parameter μ > 0, and in these cases, the maximal variance over the Gaussian’s positive support is ${\langle R_{j}\rangle }^{2}\left(\pi -2\right)/2$, which implies a maximal $K_{c}^{†}$ of π/2≈1.5708 and $P_{c,max}\approx \frac{1.57{\langle D_{i}\rangle }^{2}}{N}$. **Table 4** shows the results of statistical trials calculating rates of epitope-Ab cross-reactivity as a function of varying spread parameter, (*σ*), of the (truncated) Gaussian distribution of antibody degeneracies, given fixed repertoire size *N* and location parameter *µ*. The cross-reactivity rates closely track Var(R_j_), which here achieves a peak value of ≈ 0.452 at about *σ* =20. The rates then plateau at that of a uniform distribution (4*/*3*N*), to within discretization error.

**Table 4:** Rates of cross-reactivity of 100,000 epitopes with 100 Abs for different spreads (σ) in Ab degeneracies.

σ	⟨D_j_⟩	⟨D_j_⟩_G_	Var(D_j_)	Var(D_j_)_G_	Var(R_j_)	Rate(%)	Rate_G_(%)	Rate_R_(%)
0.25	2.00	1.55	0.000	0.002	0.000	1.000	1.001	1.000
0.50	2.00	1.66	0.000	0.022	0.000	1.000	1.008	1.000
1.00	2.00	1.87	0.000	0.070	0.000	1.000	1.020	1.000
1.25	2.06	2.15	0.116	0.296	0.027	1.028	1.064	1.027
1.50	2.16	2.31	0.294	0.419	0.063	1.065	1.079	1.063
2.00	2.48	2.70	0.890	0.935	0.145	1.144	1.128	1.145
2.50	2.84	3.09	1.694	1.622	0.210	1.211	1.170	1.210
3.00	3.17	3.44	2.344	2.244	0.233	1.245	1.202	1.245
3.50	3.56	3.85	3.486	3.322	0.275	1.277	1.224	1.275
4.00	3.96	4.25	4.878	4.569	0.311	1.312	1.253	1.311
5.00	4.71	5.01	7.540	7.189	0.340	1.354	1.300	1.354
6.00	5.47	5.76	10.795	10.327	0.360	1.376	1.325	1.374
8.00	7.11	7.34	21.351	19.361	0.422	1.438	1.373	1.437
10.00	8.71	8.84	32.286	29.794	0.426	1.400	1.354	1.398
20.00	16.14	16.20	117.620	112.183	0.452	1.453	1.427	1.452
30.00	20.73	20.91	178.354	168.686	0.415	1.402	1.372	1.401
40.00	22.93	22.97	191.807	184.996	0.365	1.352	1.337	1.351
50.00	23.62	24.01	201.720	191.451	0.361	1.349	1.319	1.348
60.00	25.13	24.60	197.318	194.524	0.313	1.368	1.376	1.367
70.00	25.53	24.97	201.208	196.203	0.309	1.337	1.342	1.335
80.00	26.00	25.21	208.000	197.218	0.308	1.309	1.310	1.308
100.00	26.00	25.49	208.000	198.331	0.308	1.309	1.305	1.308

Abs were assigned degeneracies according to a (truncated) Gaussian distribution, with varying σ parameter and fixed location parameter (µ = 0.25). Epitopes were randomly assigned to the Abs and then pairs of epitopes were randomly selected and checked for matching Ab assignments. In each trial (row), there were 100,000/(99,999x2) ≈ 5 billion tested epitope pairs. Column headings: σ – spread parameter; ⟨D_j_⟩− mean Ab degeneracy; Var(D_j_)− variance of the Ab degeneracies; Var(R_j_)− variance of the normalized Ab degeneracies; rate(%)− percentage of epitope pairs that cross-reacted with the same Ab; rate_R_(%)− percentage predicted from (Var⟨R_j_⟩ + 1)/N. ⟨D_j_⟩_G_, Var(D_j_^)G^, and rate_G_(%)–predicted mean Ab degeneracy, predicted variance of Ab degeneracies, and predicted % of pairs resulting in cross-reaction, all calculated directly from the (truncated) Gaussian distribution (see **Supplementary Material 5** for details). As σ increases, the distribution widens and ⟨D_j_⟩ rises, since µ is fixed. The number of Abs in the trials varied from N = 98 to 101 due to discretization effects, which in turn cause some small fluctuations in the actual and predicted rates.

As illustrated in **Figure 7** and discussed in the Appendix (Section 6.4), other, related unimodal distributions, such as Rayleigh, Maxwell-Boltzmann, Poisson, and uniform distributions, have similar maximal $\;K_{c}^{†\;}$values and therefore give similar results. At the other extreme, systems having widely split and skewed bimodal distributions–i.e., two sub-populations with very different population sizes and degeneracies–can have a much lower OpS, as also depicted in the figure. As described in detail in the Appendix (Section 6.2.4), a split distribution will always have a higher variance and a lower OpS than a spiked distribution. The effect is much more pronounced if the lower-degeneracy peak is much taller (and thus has a significant total probability mass). Other distributions (e.g., multimodal, less widely split/less asymmetric bimodal) give intermediate results (not shown). These facts together suggest that as long as Ab degeneracies conform approximately to Gaussian or similar unimodal distributions, the systemic probability of cross-reaction is never more than twice the minimum value, and more commonly less than ≈1.57 times the minimum value, for fixed *N* and ⟨*D_i_*⟩. Given our prior estimates for *N*, *P_c,max_* in human immunity would fall in a range of ≈ 2 ×10^–10^ to ≈ 2 ×10^–7^ for $\langle D_{i}\rangle =1,$ and 2 ×10^–14^ to ≈ 2 ×10^–11^ for $\langle D_{i}\rangle =0.01,$ with corresponding $S_{c,min}$ ranges of 1 - $P_{c,max}$.

### 3.4 Statistical trial calculations of Ab-Ag cross-reaction probabilities for varying repertoire size

As mentioned earlier and described in Methods (Section 2.3), the AEIP model was developed to predict the number of interactions between arbitrary antigens and a set of antibodies selected randomly from a larger Ab pool. Several sets of corresponding trial calculations, or phenomenological simulations, were carried out, and the results were compared to those of the model. In the main set of calculations, 10 antibodies were selected at random from Ab repertoires of varying sizes and tested against 100 billion antigens, each having 5 epitopes. For the sake of simplicity and interpretability, the model assumes that at each repertoire size, the repertoires are both complete and non-overlapping–i.e., *D_i_* =1. Hence, the model does not illustrate the effect of subtracting or adding antibodies with similar degeneracies to the immune repertoire; rather, it can be used to compare the behavior of immune systems designed with different repertoire sizes and corresponding Ab degeneracies.

**Figure 8:**
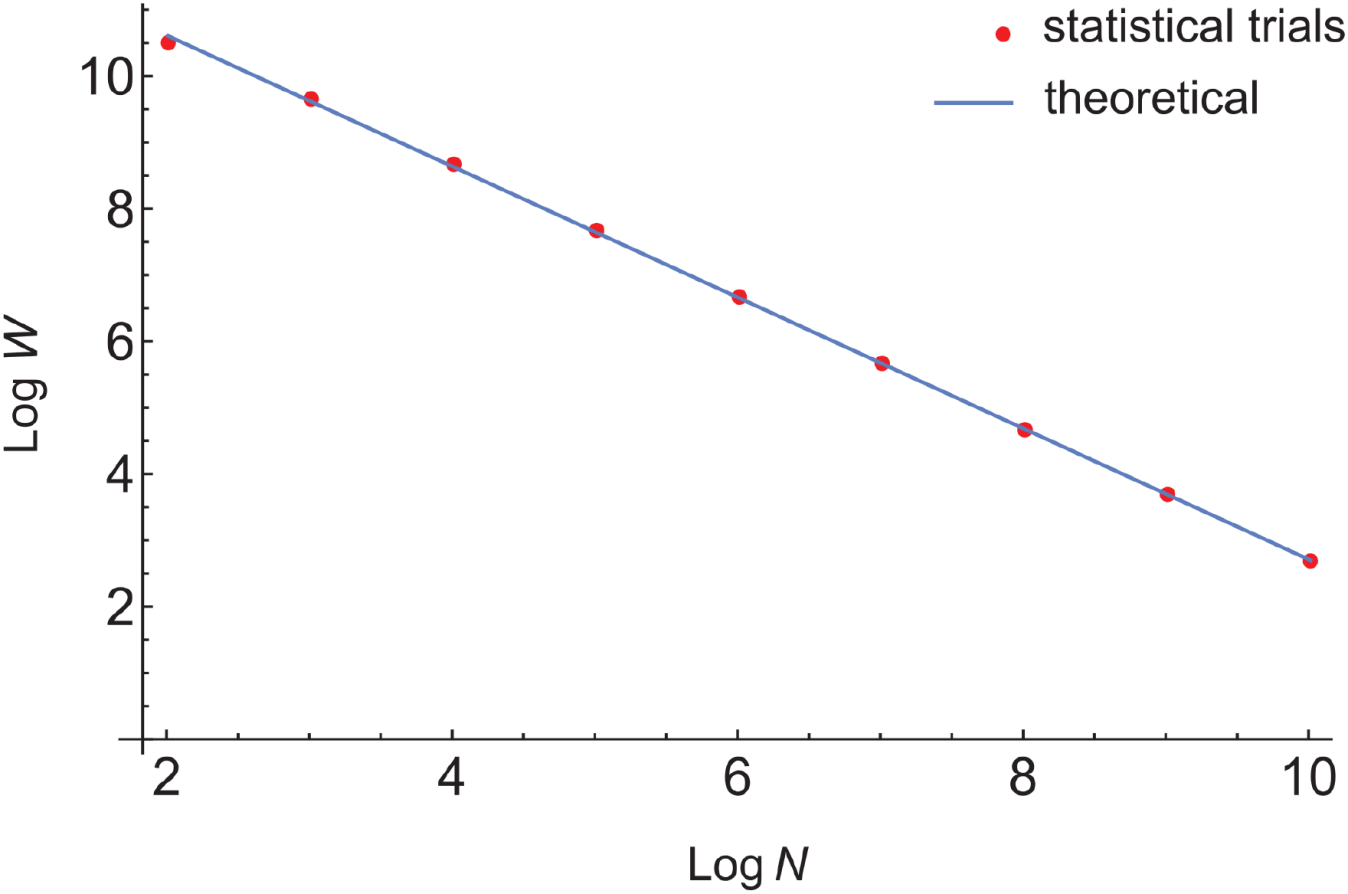
Base-10 log-log plot of the number of single, cross-reactive Ab-Ag matches between randomly selected antigens and sets of 10 antibodies selected randomly from larger repertoires (as in a polyclonal response), as a function of repertoire size. Log*W*–log of the number of antigens having complementarity to exactly one antibody in the selected set. Log*N*–log of the size of the Ab repertoire. The red dots are the trial (simulation) results, while the blue line is a least-squares-fit of the theoretical results (from the AEIP model). The number of antigens tested is 100 billion, and the number of epitopes per Ag (*ϵ*) was fixed at 5.

The results for the number of antigens having a single cross-reactive antibody match, *W*, as a function of repertoire size are shown as a log-log plot in **Figure 8**. The plot is linear, with slope -0.988, indicating that *W* drops off inversely with *N*, approximately in proportion to *N*^−0.988^. The theoretical and trial results are nearly superposable. The raw results, along with those of several approximations to the AEIP model, are given in **Supplementary Table S1** of **Supplementary Material 1.2**. For a repertoire size of *N* = 100, over 33.9% of the antigens cross-react once, whereas for *N* = 1000, only ≈ 4.8% cross-react once, with similarly decreasing results for larger *N*.

### 3.5 Anticollision (epitope cross-reaction) probability and OpS

The average epitope cross-reaction probability ⟨*P_i_*⟩ is the average probability that an epitope will be complementary to a non-cognate antibody (e.g., see dashed black arrow in **Figure 2**). It is given by $\langle P_{i}\rangle =\frac{\langle D_{i}^{*}\rangle }{N}$, where $\langle D_{i}^{*}\rangle =\langle D_{i}\rangle -1+L_{0}$ is the average non-cognate degeneracy over all epitopes–i.e., the average number of non-cognate Abs to which an epitope is complementary–and $L_{0}$ is the fraction of epitopes having degeneracy 0 (see **Supplementary Material 3.2**). Since in immunity, $\langle D_{i}\rangle$ is likely small, a Poisson distribution for the degeneracies is plausible–i.e., $L_{k}=\frac{e^{-\langle D_{i}\rangle }{\langle D_{i}\rangle }^{k}}{k!}$, where $L_{k}\;$is the probability of epitope i having $D_{i}=k$. This is because, if it is fairly rare for an epitope to be complimentary to any single antibody, then the probability of complementarity to m antibodies might be expected to fall off exponentially with m. Assuming that this is the case, $L_{0}=e^{-\langle D_{i}\rangle },$ and given our estimate of $0.01<\langle D_{i}\rangle <1$, $\langle D_{i}^{*}\rangle$ would be in a range between $\approx 5\times 10^{-5}\text{ and }0.37$.

Further, given our previous estimates for N, $\langle P_{i}\rangle$ falls in the range of $\langle P_{i}\rangle \approx 10^{-14}$ to $10^{-8}$, with $\langle S_{i}\rangle$ in the range of $\approx 1-10^{-8}$ to $1-10^{-14}$. Hence, individual epitopes would appear to be quite specific for their cognate antibodies.

However, the same is not true for systemic epitope OpS. The systemic anticollision (epitope cross-reaction) probability, $P_{a}$, is given by.

(5) $$P_{a}=\frac{{\langle D_{i}\rangle }^{2}M}{N(N-1)}\left(\frac{\text{Var}(D_{i})}{{\langle D_{i}\rangle }^{2}}+1-\frac{1}{\langle D_{i}\rangle }\right),$$

where the term in parentheses is the distribution coefficient, K_a_. Note that since *P_a_* is a sum over individual probabilities over the system, it can (greatly) exceed 1, in which case it is interpreted as the expected number of epitopes complementary to two antibodies throughout the system. For *N* ≫ 1 and a Poisson distribution of $D_{i}$, $P_{a}\;\approx \frac{{\langle D_{i}\rangle }^{2}M}{N^{2}}$ (see **Supplementary Material 3.2**). Given our previous estimates for N and $\langle D_{i}\rangle$, the systemic epitope cross-reaction probability, $P_{a}$, falls in the range of $P_{a}\approx {\left(\frac{0.01}{10^{10}}\right)}^{2}M\;$to ${\left(\frac{1}{10^{7}}\right)}^{2}M=10^{-24}M\;$to $10^{-14}M.$ Even using our conservative, lower-bound estimates for M and $M_{prot},$ this implies $P_{a}\approx \;10^{49}$ to $10^{59}$ across all epitopes and $P_{a}\approx 10^{6}\;$to $10^{16}$ across only protein/peptide epitopes. Correspondingly, the systemic OpS across all epitopes, $S_{a}\approx e^{-P_{a}}=e^{-10^{59}}$ to $e^{-10^{49}}$ (see Addendum Section 8.2.3 for derivation), which is effectively zero. Hence, the large size of these epitope spaces virtually guarantees that two randomly selected antibodies will, on average, contain many of the same epitopes in their binding spaces, although this group of shared epitopes represents only a tiny fraction (e.g., $10^{-24}$) of the total.^10^

By contrast, in the cryptographic case, $L_{0}=0$ and $\langle D_{i}\rangle =1$ (each digital file maps reproducibly to a single digital signature) and hence $\langle P_{i}\rangle =\frac{\langle D_{i}\rangle -1+L_{0}\;}{N}=0$–i.e., there are no anticollisions. Likewise, $\text{Var}\left(D_{i}\right)=0$ for SHAs, and hence $P_{a}\;=\frac{M}{N\left(N-1\right)}\left(0+1-1\right)=0$. Thus, with respect to *systemic* problem element OpS, the mathematical behavior of immune recognition and SHA functions diverges, due to the strict single-valuedness of SHAs and the size of the problem spaces. See also Discussion.

### 3.6 Average multiplicity and coverage fraction of the *H* relation

A quantity related to ⟨*D_i_*^∗^⟩ and ⟨*D_i_*⟩ is the average multiplicity of *H*, which is the average number of solution elements pointed to by each problem element in Φ_H_,


$$\bar{mult}\left(H\right)=\frac{\sum_{k=1}^{N}P\left(D_{i}=k\right)k}{\sum_{k=1}^{N}P\left(D_{i}=k\right)}=\frac{\langle D_{i}\rangle }{\left|\Phi_{\text{H}}\right|/\left|\Phi \right|}=\frac{\langle D_{i}\rangle }{f_{H}}.$$

In immunity, this is the average epitope degeneracy divided by the epitope coverage fraction. For a Poisson distribution of *D_i_*, *f*_H_ = 1 − *e*^−⟨^*^D_i_^*^⟩^, and, hence, given our estimates for ⟨*D_i_*⟩, approximate ranges for the coverage fraction and average multiplicity in immunity are $0.01<f_{\text{H}}<0.63$ and $1.005\leq \bar{mult}\left(H_{B}\right)\leq 1.58$, respectively. This means that, although a considerable fraction of all epitopes bind to at least one antibody in a given repertoire, most of the epitopes within that fraction bind to *only* one antibody–i.e., the $H_{B}$ relation is near-total and yet generally single-valued. For SHAs, $f_{H}=\bar{mult}\left(H_{K}\right)=1$.

### 3.7 Estimate of Ab cross-reactivity with self-antigens

We can estimate the expected number of antigens that will bind, or the probability of a single binding interaction, to a given (fixed) antibody. This is relevant to autoimmunity, for example. The AEIP model indicates that the probability of a single Ab-Ag interaction is linear in the number of epitopes per Ag, *ϵ* (see Methods section 2.3.1). For large *N*, the total number of interactions, can be expressed using the relation ⟨*W*⟩ = *Aϵ*⟨*P_j_*⟩, where *A* is the number of antigens accessible to an antibody. In particular, we can ask, when a new Ab is randomly generated, say by somatic mutation in the periphery, what are the chances that it will cross-react with one of the body’s own antigens? As described in **Supplementary Material 4**, a reasonable estimate for *A*, in the case of self-antigens, is 10,000, and a generous estimate for *ϵ* is 1000.

Assuming ⟨*D_i_*⟩ = 1, ⟨*P_j_*⟩ has been shown in the present study to be 10^-10^ to 10^-7^. Taking *A* ≈ to be 10,000, the average local degeneracy (i.e., “local” to a restricted set of antigens), ⟨*W*⟩ = *Aϵ*⟨*P_j_*⟩, is, therefore, in a range of about 0.001 to 1. This is the average number of self-antigens/epitopes that a single newly produced Ab species will have in its chemical binding space. Studies of polyclonal animal antibodies raised against animal proteins and tested against large arrays of human proteins have shown frequencies of strong binding events that are consistent with these statistics (110), as have studies of monoclonal Abs using panels of recombinant human antigen arrays (111). On the other hand, the lower-end estimate for ⟨*D_i_*⟩ of 0.01 results in an estimate for ⟨*W*⟩ of 10^−5^ to 10^−2^, which is somewhat lower than that expected from experiment.

### 3.8 The effect of polyclonal binding requirements on specificity

Although monoclonal Abs can elicit immune responses (112, 113), polyclonal Abs are generally more effective at activating the complement system (114, 115) and neutralizing soluble proteins or viral particles (116, 117), for example, because they more readily result in stable, multimeric Ab-Ag complexes. Thus, the probabilities with which non-cognate antigens will bind to multiple Abs, e.g., in a typical polyclonal immune response, is of interest with regard to autoimmunity. The dependence of these binding probabilities on the number of antibodies present in the response, the size of the repertoire, and the number of epitope-antibody complementarities, or matches, was explored in a set of theoretical calculations using the AEIP model, as well as a number of corresponding statistical calculations, or phenomenological simulations.

#### 3.8.1 The probability of polyclonal self-reaction.

First, consider the self-reactivity example described above, but now suppose that self-antigens are exposed to ten non-cognate antibodies instead of one. As shown in **Table 5**, the AEIP model demonstrates that the average number of self-antigens that will cross-react once (*m* =1) is, as expected, higher by a factor of ≈ 10–that is, ⟨*W*⟩ varies in a range from 0.010 to 9.99, depending on the repertoire size and assuming ⟨*D_i_*⟩ = 1.

**Table 5:** Number of self-antigens (out of 10,000 total), each having 1000 epitopes, participating in m crossreactions with 10 test antibodies selected randomly out of the total repertoire, which varies in size here from **10^7^** to **10^10^**, as calculated from the AEIP model.

m	Size of repertoire			
	10^10^	10^9^	10^8^	10^7^
0	9999.990	9999.900	9999.000	9990.004
1	0.010	0.100	1.000	9.991
2	4.495E-09	4.495E-07	4.495E-05	4.492E-3
3	1.196E-15	1.196E-12	1.196E-09	1.196E-06

However, as also shown in the table, as well as in **Table 6** and **Figure 5**, and as described analytically in the Appendix (Section 6.3), the chances that an individual antigen will participate in *m* cross-reactive interactions –i.e., *m* of its epitopes interacting with *m* distinct, non-cognate antibodies–falls off approximately exponentially with *m*. **Table 5** shows that the chances of any of the self-antigens in the prior example cross-reacting with any two of the selected antibodies is about 5 in a billion to 5 in 1000. **Figure 5** shows a log plot of the probability of *m* cross-reactive matches between any of the 10,000 self-antigens in the body and 10 antibodies selected randomly from repertoires of sizes ranging from 10^7^ to 10^10^, assuming 1000 epitopes per Ag (*ϵ* =1000). The log plot is roughly linearly decreasing with *m*, which means the probability is exponentially decreasing. Even with this high number of epitopes per Ag, the total probability that any of the self-antigens will cross-react with two of the ten selected Abs is only ≈ 4% in the smallest repertoire (*N* = 10^7^ Abs), and the chances that any will cross-react with all 10 Abs is on the order of 10^-66^ to 10^-36^ across the various repertoire sizes.

**Table 6:** Probability of cross-reactive matching for various sizes of the total Ab repertoire, N, and varying numbers of cross-reactive matches per Ag, m, assuming complete coverage of epitope space without overlap (i.e., D_i_ =1).

N	m	Trial	Exact	% Diff	% Of total
100	0	58365069993	58375236692.615	-0.0174	58.375
	1	33946511905	33939091100.358	0.0219	33.939
	2	7024474519	7021880917.315	0.0369	7.022
	3	638525059	638352810.665	0.0270	0.638
	4	25082314	25103762.217	-0.0854	0.025
	5	336210	334716.830	0.4461	3.347E-04
1000	0	95089212190	95089370457.168	-0.0002	95.089
	1	4822094044	4821976189.512	0.0024	4.822
	2	87978683	87938775.493	0.0454	0.088
	3	712467	712054.862	0.0579	7.121E-04
	4	2615	2519.911	3.7735	2.520E-06
	5	1	3.054	-67.2607	3.054E-09
10000	0	99500264643	99500899370.045	-0.0006	99.501
	1	498833733	498201979.622	0.1268	0.498
	2	900845	897930.873	0.3245	8.979E-04
	3	779	719.208	8.3136	7.192E-07
	4	0	0.252	—	2.520E-10
	5	0	3.027E-05	—	3.027E-14
10^7^	0	99999503369	99999500000.900	0.0000	100.000
	1	496628	499998.200	-0.6740	5.000E-04
	2	3	0.900	233.3341	9.000E-10
	3	0	7.200E-07	—	7.200E-16
	4	0	2.520E-13	—	2.520E-22
	5	0	3.024E-20	—	3.024E-29
10^8^	0	99999950537	99999950000.010	0.0000	100.000
	1	49463	49999.982	-1.0740	5.000E-05
	2	0	9.000E-03	—	9.000E-12
	3	0	7.200E-10	—	7.200E-19
	4	0	2.520E-17	—	2.520E-26
	5	0	3.024E-25	—	3.024E-34
10^10^	0	99999999481	99999999500.000	0.0000	100.000
	1	519	500.000	3.8000	5.000E-07
	2	0	9.000E-07	—	9.000E-16
	3	0	7.200E-16	—	7.200E-25
	4	0	2.520E-25	—	2.520E-34
	5	0	3.024E-35	—	3.024E-44

The column headings are: trial–the number of antigens, out of 100 billion tested, that cross-react m times with any of 10 antibodies selected out of the larger pool in the phenomenological simulations; exact–the results predicted from the AEIP model; % diff –the percent difference between the exact and statistical trial results ((trial-exact)/exact × 100); % of total–the number of antigens cross-reacting m times as a % of the 100 billion tested. The number of epitopes per Ag (ϵ) is fixed at 5. For a small pool of N =10 total antibodies, since all of them are selected for testing (n =10), the antigens will always cross-react at every epitope (m =5 for all). For a somewhat larger pool, N =100, the probability shifts markedly toward lower m and drops off rapidly with higher m, but there are still many antigens with multiple matches–about 7% cross-react with two antibodies and ≈ 0.6% with three. As the Ab pool becomes still larger (as in humans), single cross-reactions become less common, but multiple cross-reactive matches per antigen become very rare.

#### 3.8.2 Phenomenological simulations of multiple cross-reactions with single antigens

This general pattern of a linearly increasing probability of single cross-reactions per Ag as a function of the number of distinct antibodies in a response, accompanied by an exponentially decreasing probability of multiple cross-reactions per Ag, is also shown in a set of phenomenological simulations (see also Methods Section 2.3.2).

An increasingly large subset of antibodies (*n* =1 to 10) was randomly selected from a repertoire of fixed size (*N* =10 million) and tested against a panel of 100 million antigens, with each having 5 epitopes per Ag. **Supplementary Figure S1** in **Supplementary Material 1.1** shows the number of antigens cross-reacting once with one of the *n* selected antibodies, according to both the numerical results of the simulations as well as the exact AEIP results. (The results of 4 different approximations to the exact model, which correspond to within ±0.0004%, are given in **Supplementary Material 1.2**, **Supplementary Table S2**). Although there is some statistical variation in the numerical trial results, the overall results indicate a linear increase in the number of epitope-antibody matches as a function of the number of antibodies present (e.g., in the polyclonal response). Hence, as expected, polyclonal antibodies are likely to result in proportionately more single cross-reactive matches than a monoclonal Ab.

However, **Table 6** shows that the number of antigens, out of 100 billion, cross-reacting with *m* of the 10 antibodies selected randomly out of Ab repertoires of various sizes (*N*) decreases approximately exponentially with *m*. At larger *N*, the probability of each additional cross-reactive match drops by a factor of $\;\approx \frac{N\left(m+1\right)}{\left(5-m\right)\left(10-m\right)}$ for fixed *N*, as expected (see Appendix Section 6.3, **Equation 13**). In addition, the probabilities diminish in inverse proportion to *N*, also as expected. At a repertoire size of 10^7^, the chances of an antigen being complementary to two or more Abs are on the order of 1 in 10^11^. Hence, in humans, the probability that multiple antibodies raised in a polyclonal response would cross-react with a given non-cognate antigen, (e.g., a self-antigen) thereby triggering a potent immune response to that antigen, is normally very small.

In cryptography, the equivalent of requiring *n* Ab matches for a single antigen would be to require that the digest of an input file correspond to *n* concatenated hash values, rather than one. This would mean effectively increasing the size of the solution space of the hash function by a factor of *n*, e.g., from SHA-256 to SHA-512, which exists as part of the SHA-2 standard (118, 119), or SHA-1024, which does not.

## 4 Discussion

This study has described the statistics that underlie the human immune system’s paradoxical ability to recognize an extremely large set of possible antigens (Ags) while retaining apparent specificity for particular cognate antigens. As has been illustrated, immunity accomplishes this by using strategies that mathematically parallel those used by cryptographic hash functions such as SHA-256. Both systems employ solution elements (antibodies, hash values) that are, at least on average, highly degenerate or multispecific toward their problem elements (epitopes, digital files), yet appear to maintain specificity for their originating or primary problem elements in real-world operation. Moreover, the study illustrates in a quantitative, albeit approximate, manner why multispecificity and specificity are viewed most usefully not as different points along the same parameter axis, but as distinct parameters or properties with different, though related, mathematical forms. In particular, specificity is a function of the degree of multispecificity, as well as other system variables.

### 4.1 Antibody degeneracy

The large size of epitope space, together with the need for completeness of antigen recognition, implies that antibodies must have high binding degeneracies, at least on average. This is a straightforward application of the pigeonhole principle (120) to humoral immunity. Other authors have pointed out that T-cell receptors must be multispecific (25, 38), because of the large number of possible presenting peptides. In 1998, Mason estimated that one T-cell can respond to 10^8^ different 11-mer peptides, and T-cell multispecificity has been experimentally confirmed (29). Multispecificity, or degeneracy, has also been understood to be a property of at least some antibodies (26, 33, 39, 40, 121–124). It is well-known that a single Ab variable region can have within it multiple distinct binding sites or paratopes (125), or different paratope states (27, 126, 127), that bind completely different epitopes. A single Ab paratope can bind different, unrelated epitopes (128–130), or different epitopes on the same Ag (59). Germline or “natural” antibodies–those found in human serum in the apparent absence of antigenic stimulation and which are primarily of the IgM class–are known to be “polyreactive” (26, 121), although often with low affinity. Conventionally, it has been believed that the binding regions of polyreactive antibodies tend to be more flexible (123, 131, 132), although there is evidence against this (133, 134), and a 2020 analysis indicated that polyreactive antibodies also tended to be less strongly negatively charged and less hydrophilic, while tending to have longer CDR loops in the heavy chain (135). In general, however, antibodies, and particularly affinity-matured antibodies (41, 136–139), are believed to be more specific than T-cell receptors. Overall, it has remained unclear as to how antibody multispecificity should be interpreted in the context of cases in which antibodies demonstrate exquisite specificity for particular antigenic targets.

Moreover, a global, systematic, quantitative analysis of human antibody degeneracy and its relation to specificity has not been previously undertaken. Some authors have characterized the number of possible, distinct antigens as “infinite” (39, 40). Here, through straightforward modeling and the use of prior experimental data, we arrive at conservative lower-bound estimates for the number of possible, hapten-related epitopes and protein/peptide epitopes of *M* =10^83^ and *M_prot_* =10^30^, respectively.^11^ These results imply a conservative, lower-bound estimate for the average degeneracy of antibodies to be ≈10^71^ epitopes, of which at least ≈10^18^ represent protein or peptide epitopes. Hence, *H_B_*, the relation which takes epitopes to antibodies in an individual repertoire, is very highly *many-to-one*, at least on average.

The cryptographic case is similar: hash functions must be capable of handling any of an enormous number of possible digital files–far greater, even, than the number of possible epitopes. For a 4000-bit digital file space (roughly 100 English words), this number is *M* ≈ 10^1204^, which implies an average hash value degeneracy of ≈ 10^1127^ files or messages. Thus, as is known, the hash values generated from, and assigned to, input digital files as distinguishing markers are, in fact, not at all specific in an absolute sense (140–143). In this same sense, antibodies, at least on average, are far from being absolutely specific to their cognate epitope or antigen.

### 4.2 The specificity paradox

The specificity paradox is that, despite this necessary degeneracy, multispecificity, or “promiscuity”, antibodies often appear to be specific to their cognate antigens in laboratory testing or clinical use (144–146), and hash functions such as SHA-256 are, in practice, highly effective digital security tools. The explanation is that the utility of these systems depends not as much on absolute specificity as it does on the degree of specificity. This idea, expressed in other terms, is well known in cryptography, but it is not widely appreciated for antibodies. In the immunological literature, the notion of polyspecificity, multispecificity or degeneracy has often been conceived of as a sort of opposite of specificity, implying a many-to-one relationship as opposed to a one-to-one relationship. This has led to some confusion.

Degeneracy is the number of complementary partners an element has in a relation–e.g., the number of epitopes to which an antibody is complementary. By contrast, specificity, strictly defined here as *operational* sp*ecificity*, is an element’s unlikelihood of being complementary to an arbitrary, non-cognate partner–e.g., of an antibody’s being complementary to a non-cognate epitope. Hence, an element in a relation can be both highly degenerate–i.e., highly multispecific–with respect to its possible partners and, simultaneously, highly specific, without contradiction.

As described by the models and simulations in this work, the average solution element OpS is very high in both types of systems: ≈ 1-10^-77^ for SHA-256, and ≈ 1-10^-7^ to 1-10^-12^ for the human antibody repertoire.

Hence, the solution elements in either system are sufficiently large, non-overlapping, and, as discussed below, uncorrelated to exhibit the specificity required for them to work as intended in their contexts of use. Although an Ab recognizes many molecular structures, those structures are scattered throughout chemical space and the binding repertoire. Thus, as proposed by prior authors for T-cell receptors (28), the probability that a given Ab will recognize a single, randomly selected antigen or epitope is still low. The same holds true for digital files and hash values (141, 143, 147). Since the probability of collisions or cross-reactions varies inversely with solution repertoire size, *N*, repertoires in these systems must be large enough to make those events sufficiently rare, yet small enough to be feasible. In addition, because the average and systemic cross-reactive probabilities $\langle P_{j}\rangle$ and $P_{c}$, depend on $\langle D_{j}\rangle /M$ and ${\langle D_{j}\rangle }^{2}/M^{2}$, respectively, it is true that as epitope spaces increase in size (*M*), the cross-reactive probabilities and corresponding OpS’s remain constant so long as the antibody degeneracies, ⟨*D_j_*⟩, grow proportionately– and they do if epitopes are distributed randomly across the antibody binding spaces. Similarly, in cryptography, doubling the digital file size (squaring *M*) does not change the average OpS of a hash value, since ⟨*D_j_*⟩ increases proportionately (by a factor of *M*). On the other hand, when the size of the solution space, *N*, increases, the average specificity rises, presuming the problem element degeneracy, ⟨*D_i_*⟩, is fixed.

The meaning of systemic OpS differs substantially from that of individual OpS or its average across the system. For solution elements (e.g., antibodies), the latter two quantities are measures of whether a randomly chosen problem element (e.g., epitope) is likely to be complementary to a particular solution element. Systemic OpS, by contrast, measures how improbable it is for a collision or cross-reaction to occur anywhere across the entire system. For high average solution element degeneracies (⟨*D_j_*⟩ ≫ 1), it has been shown here that the systemic probability of collision varies approximately as $\;P_{c}\approx \frac{{\langle D_{i}\rangle }^{2}}{N}\left(\text{Var}\left(R_{j}\right)+1\right)$, where *R_j_* is the degeneracy normalized to the mean. For small individual collision probabilities (i.e., large spaces), the systemic OpS is, then, *S_c_* ≈ *e*^−^*^P_c_^*, which reduces to 1 − *P_c_* when *P_c_* is ≪ 1, as it is for antibodies in a human immune repertoire or hash values generated by an SHA.

In the case of SHA functions, since all *D_i_* =1, and the spread of hash value degeneracies (Var(*D_j_*) or Var(*R_j_*)) is effectively 0, these collision probabilities and the associated OpS’s are likely very close to the solution element average (1-10^-77^), which is the minimum possible value for fixed, large *N*. Less is known about the distributions of antibody degeneracies, but there is some evidence that they are approximately Gaussian. The current work, together with the results of prior studies on statistical distributions (107–109), has illustrated that, for a fixed repertoire size and average epitope degeneracy, ⟨*D_i_*⟩, Gaussian and many other unimodal, Gaussian-like distributions give rise to maximal cross-reaction probabilities of, at most, twice that of the minimum, and more often less than ≈ 1.57 times the minimum. Hence, systemic OpS is rather insensitive to shifts within and between these kinds of unimodal degeneracy distributions, for a large, fixed repertoire size. By contrast, a system with a widely split, bimodal degeneracy distribution and a tall left-hand peak, for example, would have a specificity that is significantly lower, by virtue of an increased variance, than that of the optimal configuration. All of this would seem to apply to hash values as well.

In humoral immunity, the current work has illustrated that, as expected, the average epitope degeneracy–i.e., the average number of distinct Abs capable of binding a particular epitope, ⟨*D_i_*⟩–increases linearly with the number of Ab species available, assuming the average size of the individual Ab binding spaces is constant. Further, the quadratic dependence of the probability of Ab cross-reactivity, *P_c_*, on ⟨*D_i_*⟩, underscores why it is important for the immune system to have as low a ⟨*D_i_*⟩ as possible while still ensuring, statistically, that the immune response will recognize multiple epitopes on any arbitrary antigen.

A related, unforeseen result of this analysis has been that while the average epitope OpS is fairly high–estimated here to be ≈ 1 − 10^−14^ to 1 − 10^−8^, the *systemic* epitope OpS, *S_a_*, is effectively zero. This arises from the fact that for large problem/solutions spaces, systemic OpS is *S_a_* ≈ *e*^−^*^Pa^* and that, in contrast to the case of antibody cross-reactions or collisions, the probability (or, here, the number) of anticollisions–i.e., the expected number of epitopes complementary to any two randomly selected antibodies– $P_{a}\;\approx \frac{{\langle D_{i}\rangle }^{2}M}{N^{2}}\left(\frac{\text{Var}\left(D_{i}\right)}{{\langle D_{i}\rangle }^{2}}+1-\frac{1}{\langle D_{i}\rangle }\right)$ is very high in absolute terms. For protein or peptide epitopes, it is at least 10^6^ − 10^16^, and for the set of all epitopes, far higher. In this way, the immune system diverges from SHAs, for which *P_a_* = 0 and *S_a_* = 1. This occurs because, although the prefactor $\frac{{\langle D_{i}\rangle }^{2}M}{N^{2}}$ is extremely large in both systems due to the large problem spaces, the distribution coefficient $\left(\frac{\text{Var}\left(D_{i}\right)}{{\langle D_{i}\rangle }^{2}}+1-\frac{1}{\langle D_{i}\rangle }\right)$ for SHA digital file degeneracies is 0, while that for epitope degeneracies is *>* 0 (and equal to 1 for a Poisson distribution). Hence, while an individual epitope will, on average, be very specific for its cognate antibody in an immune repertoire, chemical/epitope space is so large that two antibodies selected randomly from that repertoire will still be statistically guaranteed to be complementary to many of the same epitopes, although the fraction of such epitopes relative to the total is extremely small (10^−24^ to 10^−14^).^12^ We have thus demonstrated that in humoral immunity, the *H_B_* relation is almost certainly multivalued over at least part of its domain, but that this “multivalued-ness”, or multiplicity, is limited, estimated here to be ≈ 1.005 to 1.58 antibodies per epitope, on average, assuming a Poisson distribution of epitope degeneracies. Along those same lines, the average non-cognate degeneracy of an epitope is estimated to be only ≈ 0.00005 to 0.37 antibodies. Thus, while it appears that potential anticollisions (epitope cross-reactions) must exist in humoral immunity in large numbers, they are expected to actually occur fairly infrequently as long as epitopes are randomly distributed across antibody binding space.

This helps demonstrate that although the *H_B_* relation does not mirror cryptographic hash functions exactly, in that it is not exactly a total, single-valued function, it does approximate one. The average epitope degeneracy, ⟨*D_i_*⟩, which is a measure of the coverage fraction of *H_B_*–i.e., the number of epitopes recognized by an individual’s repertoire (Φ_H_) relative to the number of all possible epitopes (Φ)– while not exactly 1, is likely within an order of magnitude or two less than one. In fact, assuming a Poisson distribution, it implies a coverage fraction of ≈ 0.01 to 0.6, which is remarkably high, given the size of Φ (*M* or *M_prot_*). Interestingly, calculated rates of Ab cross-reactivity assuming ⟨*D_i_*⟩ = 1 were more consistent with experiment here than those assuming ⟨*D_i_*⟩ = 0.01 (Results Section 3.7). At the same time, *H_B_* is not highly multivalued, as just mentioned. The human immune repertoire thus seems to have been evolutionarily tuned in size and specificity to cover all of antigenic space in any single individual without much redundancy in epitope binding. If ⟨*D_i_*⟩ were higher, for example, there would be more cross-reactivity, and if it were lower, immune recognition might be incomplete–i.e., antigenic totality might not hold.

### 4.3 Random association

One might argue, especially with regard to antibodies, that the specificity for local changes in epitope structure is significantly worse than the above estimates would imply, because local changes in antigen structure may not, in some cases, produce large changes in Ab binding affinity (148). It is true that immunological cross-reactions are more probable in nearby (82) than more distant (110, 111) regions of chemical/epitope space. Finding cross-reactivity is easier among related drug molecules (149), homologous antigens across species (150, 151), or surface antigens in different strains of a virus (152), for example, than it is among distantly related or unrelated antigens. This effect is mirrored by some types of cryptanalytic attacks, such as differential attacks, which exploit the fact that collisions are more apt to be found through local perturbations of digital messages than through large changes (153–155). It is also true that, over the course of an individual’s lifetime, there will be constant modification of the Ab repertoire due to affinity maturation and the filtering out of self-reactive antibodies (156, 157), so that some non-randomness is introduced (158).

However, because of 1) the randomness involved in immune gene recombination (16, 17) described earlier, 2) avalanche-type effects in Ab-Ag structure-affinity relationships (96–101), which, while often not as great as those in SHA functions, are still significant, and 3) the sheer size of chemical space, the vast majority of epitopes to which the immune system is naïve will still be more-or-less randomly distributed across Ab variable regions. This is the immunological equivalent of hash functions generating pseudo-random hash value output for each unique digital message input (92, 159).

In this way, degeneracy and specificity are decoupled in these systems. As long as hash functions generate random output, they can take on digital messages of arbitrary length (increases in *M*) without any significant loss of OpS in their hash values–what in cryptography is known *collision resistance (*160). Similarly, in adaptive immunity, as long as there are no correlations between new epitopes and, for example, antibodies/cell receptors directed against self-antigens, the immune system can afford complementarity to any arbitrarily large number of different, random epitopes without incurring higher rates of cross-reactivity. In the case of some autoimmune diseases, epitopes on pathogens can “mimic” self-epitopes such that their cognate antibodies or cell receptors are very likely to cross-react with self (161, 162) and thus the normal statistics do not hold. In a similar way, correlations between new inputs and target hash values “break” an SHA, which means they negate its security or utility (155). Hence, in both systems, randomness is a key design feature–not just to create diversity in the solution space, but to create uncorrelated diversity.

### 4.4 Affinity maturation and absolute specificity

Affinity-matured antibodies have a higher affinity for their cognate antigens because of the diversification and amplification of selected combining site populations that occurs during the maturation process (163, 164). One line of thinking has been that these antibodies are also more specific than primary or germline antibodies (136–139), possibly because they are more rigid (165–168). However, other studies indicate that the higher affinity may arise from a number of mechanisms unrelated to flexibility (166, 169–171).

In either case, the body’s ability to respond effectively to an antigen to which it is naïve depends critically on the diversity that exists prior to the initiation of the affinity maturation process with respect to that particular antigen. An immune repertoire that has undergone many affinity maturation events must still retain sufficient degeneracy to respond to any arbitrary antigens in the context of a limited, albeit large, total number of immune receptor species. As illustrated in this work, and as mentioned above, an immune repertoire with a bimodal distribution of antibody degeneracies has a lower systemic operational specificity than one with a singular distribution, more so if the split in the distribution is wide, with the taller peak at lower degeneracies. Not surprisingly, even antibodies that have undergone affinity maturation have been shown to cross-react with both related (80–83, 110, 149, 172, 173) and unrelated epitopes (110, 111, 128, 129, 174–177).

These findings and the statistics of immune receptors and molecular diversity as detailed in this work and as paralleled by the statistics of SHA collisions, would suggest that cross-reactive antigens to any antibody, affinity-matured or primary, probably exist somewhere in chemical/peptide space, although they may be difficult to find. Experimental data on the size of the binding spaces of individual antibodies is currently scarce, and nothing in the present work rules out the existence of particular antibodies that are absolutely specific to their cognate epitopes. However, it appears to be highly statistically, chemically and functionally improbable. Terms like “monospecific” or “monoreactive” should be understood in this context.

### 4.5 Factors limiting cross-reactions and collisions

As also illustrated in the current work, factors that reduce the number of cross-reactions or collisions are 1) restriction of the effective problem domains, 2) multiple-match requirements (at least in the case of immunity), and 3) low-variance degeneracy distributions, which were discussed above. In real-world operation, the absolute number of collisions or cross-reactions depends not only on the antibody specificity, which is essentially a ratio or “rate,” but on the number of problem element inputs with which the system will actually be presented, which is generally much smaller than the set of all possible inputs. As described in **Supplementary Material 6**, the average person will be exposed to an extremely small fraction of all possible antigenic molecular structures over his/her lifetime, and the number of self-antigens to which a novel Ab will be exposed is also relatively small. Similarly, cryptanalysts, Bitcoin miners, and thieves are limited in their searches for collisions by computational capacity and cost.

As to multiple-match requirements, this analysis illustrates the statistics by which the linkage between polyclonal antibody binding and a potent immune response likely boosts operational specificity for whole antigens relative to individual epitopes. Although there are exceptions, a potent immune response generally requires the binding of multiple antibodies to an antigen and the formation of immune complexes (see ref (178) for a review). Because they bind to different epitopes on the antigen, polyclonal antibodies facilitate the formation of these complexes. They are commonly thought of as being less specific than monoclonal antibodies (179–181), and this is true, as measured by their collective degeneracy. As illustrated in the current work, a set of multiple, distinct antibodies will have a proportionately larger antigenic binding space than an individual (monoclonal) Ab. For this reason, it has been surmised by some that polyclonal immune responses may contribute to autoimmunity (182). However, as is also illustrated here, the probability that the same antigen (e.g., a self-antigen) will cross-react with several non-cognate antibodies raised in an immune response is low, dropping off exponentially with the number of epitope-antibody matches. Because the binding spaces of the constituent antibodies in a polyclonal response are (randomly) different, the likelihood of a non-cognate antigen cross-reacting with several of them is approximately the product of the individual likelihoods. In this way, the requirement that the immune system imposes on a given antigen to participate in multiple Ab-Ag interactions before allowing it to trigger a potent immune response very likely helps to prevent autoimmunity and other non-targeted responses. The mechanism may be likened to multi-step authentication in digital security.

### 4.6 Other parallels and potential applications

There are other parallels between adaptive immunity and cryptology that have not been mentioned in this analysis. In some cases, these cross-disciplinary connections may provide insights or suggest avenues of investigation.

For example, although SHAs are often modeled as random oracles (92), and although their outputs are in fact close to randomly uniform distributions, cryptanalysts exploit deviations from uniformity in many types of attack by localizing target hash values to more highly populated regions of the hash value domain (e.g., references (155, 183)). In a similar way, recombination in immune cell receptor genes is not entirely random and uniform (184), and rates of somatic hypermutation, a genomic process that occurs as part of affinity maturation, also show some location- (21) and sequence-related (185, 186) biases. It is known that in B-cells that are not naïve to antigens, the heavy chain tends to determine the light chain, a phenomenon called light chain coherence (158). It is not yet clear whether any of these deviations from randomness also result in non-uniformity in the Ab binding repertoire as it affects the coverage of antigenic space; presumably, they may. Through antigenic drift (187–189) and shift (190), pathogens like viruses and bacteria mutate or genetically reassort to evade the adaptive immune response. However, the extent to which they may “attack” binding repertoire non-uniformity–i.e., occupy or mutate into regions of epitope chemical space whose cognate antibodies reside in “cold spots” in their coding sequences–has not yet been well explored and represents a potential area of research. Additional parallels between adaptive immunity and cryptography, some of which suggest other avenues of inquiry, are discussed in **Supplementary Material 7**.

Finally, the analytic framework developed here and its future extensions and refinements may have applications in predictive calculations–for example, in quantitative predictions of cross-reactivity among sets or pools of antibodies (191–193), particularly as more data is collected with respect to immunologic parameters such as antibody binding space sizes and epitope degeneracies. Knowledge of the mathematical relationships among system parameters should enable the determination of any single parameter, given data on the others (e.g., by rearrangement of Equation 4), or, when data on all the parameters is available, enable checks on their mutual consistency.

## 5 Conclusion

This study has used a probabilistic systems analysis approach to describe the statistics that underlie human antibody-antigen complementarity. It has provided conservative, lower-bound, order-of-magnitude estimates for antibody degeneracy, or multispecificity, while also defining, formulating, and quantifying the concept of operational specificity. It has illustrated why the degeneracy of human antibodies must be extremely high, at least on average, and that the properties of degeneracy and operational specificity (OpS) are distinct and, in an important sense, decoupled: as long as the assignment of epitopes to antibodies–i.e., the *H_B_* relation–is random, OpS remains constant as the size of epitope space varies. This helps to explain and quantify the specificity paradox–namely, that antibodies can be highly degenerate, or “multispecific,” in their binding to epitopes and still display significant clinical and laboratory specificity. In particular, antibodies are specific enough for the body to be able to tolerate the production of new ones, given the number of self-antigens that they are likely to encounter, and given that the binding of cognate and non-cognate epitopes is generally uncorrelated. In addition, it has been shown here how the immune system’s imposition of multi-epitope recognition requirements, executed *via* the polyclonal response, increases specificity and likely helps avert autoimmunity.

The present study has also illustrated that adaptive immunity shares many similarities with cryptographic hash algorithms in its organization and function. The digital fingerprints produced by hash functions such as SHA-256 are even more highly degenerate than antibodies, but they are also more operationally specific, because of the greater size of their solution spaces, again illustrating how the two properties are uncoupled. Further, *H_B_* approximates the behavior of SHAs, which are total, single-valued functions, by being near-total while managing to avoid high multiplicity. The parameters in humoral immunity have apparently been “tuned” to statistically ensure that multiple epitopes will be recognized on an arbitrary antigen, while minimizing the chances that any epitope will be recognized by multiple antibodies.

This work is intended as a first attempt at formalizing the analysis of degeneracy and specificity in these types of systems; it is expected that the analysis will be extended in the future to include Ψ^C^, the set of all possible human antibody species, and that the numerical estimates will improve. By delineating the relationships between system parameters involved in humoral immunity, the current models extend our understanding of the statistics of cross-reactivity and could contribute to predictive calculations. The parallels between immunity and cryptography may suggest cross-disciplinary research.

## Data Availability

The original contributions presented in the study are included in the article/**Appendix**/**Supplementary Material**. Further inquiries can be directed to the corresponding author.

## Footnotes

1 Here, we extend the notion of *cognate*, which denotes a primary (or causal) and unique pairing between an epitope and an antibody, to the general case of any system containing problem and solution elements that can be identified as having such a relationship, including cryptographic hash systems.

2 The same is true for “antibodies”, which refers to a set of unique antibody species–more specifically, unique variable regions, as well as the terms “solution elements”, “problem elements”, “hash values”, and “digital files”.

3 It is highly probable that all (fully formed) antibodies bind at least one epitope. Similarly, all hash values are thought to have at least one possible originating file, although this has not been proven.

4 The *degree* of the element, in set theory. This is similar to the preimage cardinality of the element under *H* but degeneracy (or degree) is a property that extends to all domain or codomain elements, including those with degeneracy 0.

5 The units here are *problem elements*, or, e.g., *epitopes*, but we will ignore units for conciseness in most of this work.

6 Technically, a *second preimage*, in cryptography.

7 Specificity =1−(false(+)/cond(−)), where “ false(+)” is the number of individuals in a population testing falsely positive and “ cond(−)” the number who do not have the illness or condition.

8 As mentioned, the “*i*” subscripts here always correspond to problem elements (e.g., epitopes) and the “*j*” subscripts to solution elements (e.g., Abs).

9 This is significantly less than the upper limit of diversity that can, in theory, arise from immune cell gene recombination (21, 194, 195), illustrating that only a small fraction of total possible antibody and receptor variable region diversity (Ψ^C^) is realized in any one individual (Ψ).

10 i.e., a *single* epitope would cross-react with one of these randomly selected antibody pairs very infrequently, provided epitopes are randomly distributed throughout the Ab binding space.

11 Since there are ≈10^32^ total (i.e., not unique) protein molecules in the entire human population (196) and ≈10^80^ atoms in the known universe (197), this illustrates that only a small fraction of all possible species of epitopes or antigens are ever instantiated. Still, there is no evidence to suggest the immune repertoire would fail to recognize any of them.

12 This is also the approximate probability that two randomly selected epitopes will cross-react with a given antibody, since ⟨*D_i_*⟩^2^*/N*^2^ = ⟨*D_j_*⟩^2^*/M*^2^.
