# Correction: Antibodies and cryptographic hash functions: quantifying the specificity paradox

**DOI:** 10.3389/fimmu.2025.1756946

**Published:** 2026-01-05

**Authors:** Robert J. Petrella

**Affiliations:** 1 Department of Chemistry and Chemical Biology, Harvard University, Cambridge, MA, United States; 2 Harvard Medical School, Boston, MA, United States

**Keywords:** antibodies, adaptive immune system, receptors, antigens, epitopes, degeneracy, polyspecificity, polyreactivity

In the **Introduction**, the first sentence under point #5 contains a single instance of the word “antigens”, which should be replaced with “antibodies”. A correction has been made to the section **Introduction**, paragraph 5 as follows:

“5. The specificity of individual epitopes for their cognate antibodies is quite high: in the range of 1−10^−14^ to 1−10^−8^, but epitope space is so large that it virtually guarantees, statistically, that two randomly chosen antibodies in an immune repertoire will share many common epitopes in their binding spaces–conservatively, ≈ 10^6^ to 10^16^ protein or peptide epitopes, on average (Results Section 3.5), although this is a very small fraction of the total size of the relevant epitope space.”

The original version of this article has been updated.

