# Supplementary material for: Antibodies and cryptographic hash functions: quantifying the specificity paradox
Source: Front Immunol. 2025 Nov 5;16:1585421. doi: 10.3389/fimmu.2025.1585421 (PMC12627013; doi:10.3389/fimmu.2025.1585421)
Supplement: Supplementary file 1 [file DataSheet1.pdf]

Antibodies and cryptographic hash functions: quantifying the specificity paradox

Robert J. Petrella [1,2,*]

[1] *Department of Chemistry and Chemical Biology, Harvard University, Cambridge, MA 02132,* [2] *Harvard Medical School, Boston, MA 02115*

Correspondence*:
petrella@fas.harvard.edu, robertjpetrella@yahoo.com

6 APPENDIX

6.1 The PECS model's underestimate of total protein/peptide diversity

The PECS model very likely underestimates total peptide/protein epitope diversity for the following reasons. First, an interface size of q residues does not necessarily imply only q possible (x, y) positional "slots" for amino acids in a real epitope. For example, if we considered 30 possible (x, y) positions for q =15 residues, the estimate would increase by a factor of 30!/(15!15!), or about 155 million. However, here we are limiting the number of slots to the number of residues. Second, in real systems, each amino acid can also adopt different spatial orientations. In particular, for non-Gly residues, the Cα-Cβ bond of the side chain can point in different directions. Third, for a given set of Cα positions and Cα-Cβ bond orientations, there can be many different sets of side chain orientations, particularly for large residues such as Lys, Arg, Glu, Gln, Met, and Trp. A geometric mean of only three different side chain orientations per residue would imply over 14 million unique sets for a 15-residue epitope, many of which would represent different epitope structures under our definition. In this work, all of these contributions are being ignored, because we are seeking a lower bound estimate for the number of unique structures.

6.2 Derivations for degeneracies and operational specificities

6.2.1 Individual solution elements

If ${D_j}^*$is the *non-cognate degeneracy* of solution element j, or the number of non-cognate problem elements to which it is complementary, then the probability, P_j, that a randomly selected problem element will be non-cognate to j is

$$P_j = \frac{{D_j}^*}{M} = \frac{D_j - 1}{M}, \tag{6}$$

for integer $D_j \geq 1$.

Then, $P_j = \frac{R_j \langle D_j \rangle}{M} - \frac{1}{M} = \frac{\langle D_i \rangle}{N} \left(R_j - \frac{1}{\langle D_j \rangle} \right)$, where R_j is the normalized degeneracy. The specificity of solution element j is then $S_j = 1 - \frac{D_j - 1}{M} = 1 - \frac{\langle D_i \rangle}{N} \left(R_j - \frac{1}{\langle D_j \rangle} \right)$.

For $\langle D_j \rangle \gg 1$, the probability and OpS can be approximated as $P_j \approx \frac{D_j}{M} = P_{0j}$ and $S_j \approx 1 - \frac{D_j}{M}$.

6.2.2 Averages over the system

For the average solution element probability, again assuming all D_j are integers ≥ 1,

$$\langle P_j \rangle = \frac{1}{N} \sum_{j=1}^{N} \left(\frac{D_j - 1}{M} \right) = \frac{\langle D_j \rangle - 1}{M} = \frac{\langle D_i \rangle}{N} \left(1 - \frac{1}{\langle D_j \rangle} \right). \tag{7}$$

The average solution specificity across the system is correspondingly $\langle S_j \rangle = 1 - \frac{\langle D_j \rangle - 1}{M}$.

And if $D_j \gg 1$, $\langle P_j \rangle \approx \frac{\langle D_j \rangle}{M} = \frac{\langle D_i \rangle}{N}$, and $\langle S_j \rangle = 1 - \frac{\langle D_j \rangle}{M} = 1 - \frac{\langle D_i \rangle}{N}$.

Note that these average quantities depend on the mean degeneracies across the system (not on higher-order moments) and are independent of any existing statistical correlations.

In the case of small $\langle D_j \rangle$, e.g., $\langle D_j \rangle < 1$, $\langle P_j \rangle = \frac{\langle D_j \rangle - 1 + L_0}{M}$, where L_0 is the fraction of problem elements with a degeneracy of 0. (See Supplementary Material 3).

In the AEIP model (described Methods Section 2.3.1), in which the selection of Abs and Ags is random, if the number of selected antibodies (n), epitopes per Ag (ε), and matches (m) are all equal to 1, the probability of interaction, $P(1, 1, 1, N) = (N - 1)!/N! = 1/N$, corresponding to the result above, since $\langle D_i \rangle$ in the AEIP model is fixed at 1.

6.2.3 Systemic probabilities and OpS

We define the *systemic OpS* on the basis of the systemic collision probability, which is the probability of a cross-reaction occurring anywhere in the system, including the initial cognate pair interaction and a second non-cognate interaction.

Systemic collision probability

Provided that H is random– e.g., that epitope-antibody complementarity is uncorrelated–the probability that a randomly selected problem element ϕ_{i_1} will point to element ψ_j, and thus establish a cognate pair, is $P_{i_1 j} = \frac{D_j}{M}$. The probability that a second randomly selected problem element (which is, by definition, non-cognate) $\phi_{i_2 \neq i_1}$ will also point to ψ_j is $P_{i_2 j} = \frac{D_j - 1}{(M-1)}$. Hence, the total probability of a collision between $(\phi_{i_1},\ \psi_j)$ and $(\phi_{i_2},\ \psi_j)$ is $P_{i_1 i_2 j} = \frac{D_j (D_j - 1)}{M(M-1)}$. This is only for a single solution element ψ_j. Then, considering cognate pairs formed with any of the N solution elements in the entire system, the probability[1] of a collision between a solution element and one non-cognate problem element, P_c, is:

$$P_c = \sum_{j=1}^{N} P_{i_1 i_2 j} = \sum_{j=1}^{N} \frac{D_j (D_j - 1)}{M(M-1)} = \frac{1}{M(M-1)} \left(\sum_{j=1}^{N} {D_j}^2 - \sum_{j=1}^{N} D_j \right). \tag{8}$$

Since $\mathrm{Var}\,(D_j) = \frac{1}{N} \sum_{j=1}^{N} {D_j}^2 - \langle D_j \rangle^2$, it is clear that $\sum_{j=1}^{N} {D_j}^2 = N \left(\mathrm{Var}\,(D_j) + \langle D_j \rangle^2 \right)$, and since $\sum_{j=1}^{N} D_j = N \langle D_j \rangle$,

$$P_c = \frac{N}{M(M-1)} \left(\mathrm{Var}\,(D_j) + \langle D_j \rangle^2 - \langle D_j \rangle \right).$$

[1] As mentioned, the interpretation of P_c as a probability only holds when $P_c < 1$. Otherwise, it can be interpreted as an expected count of collisions or cross-reactions across the system. The systemic (anti)collision probability, P_a, for epitopes across a human immune repertoire does, in fact, greatly exceed 1 (see Results Section 3.5).

Factoring out $\langle D_j\rangle^2$,

$$P_c = \frac{N\langle D_j\rangle^2}{M(M-1)}\left(\frac{\mathrm{Var}\,(D_j)}{\langle D_j\rangle^2}+1-\frac{1}{\langle D_j\rangle}\right) = \frac{N\langle D_j\rangle^2}{M(M-1)}\left(\mathrm{Var}\,(R_j)+1-\frac{1}{\langle D_j\rangle}\right) =$$

$$\frac{\langle D_i\rangle^2}{N}\frac{M}{(M-1)}\left(\mathrm{Var}\,(R_j)+1-\frac{1}{\langle D_j\rangle}\right), \tag{9}$$

where $K_c = \left(\mathrm{Var}\,(R_j)+1-\frac{1}{\langle D_j\rangle}\right)$ is called the *distribution coefficient* for solution element j, and $K_c \geq 0$, for integral D_j. This expression is derived without assumptions about the relative magnitudes of $N,\ M,\ \langle D_i\rangle$, and $\langle D_j\rangle$, except that $D_j \geq 1$.

For $M \gg 1$ and $\langle D_j\rangle \gg 1$, as is the case in the current analysis, and using $\langle D_i\rangle = \langle D_j\rangle N/M$, this reduces to

$$P_c \approx \frac{\langle D_i\rangle^2}{N}\left(\mathrm{Var}\,(R_j)+1\right) = \frac{\langle D_i\rangle^2 K_c^\dagger}{N} = \frac{\langle D_j\rangle^2 N K_c^\dagger}{M^2}, \tag{4}$$

where $K_c^\dagger \approx \left(\mathrm{Var}\,(R_j)+1\right)$ is the "high-mean" distribution coefficient.

Systemic OpS

The systemic operational specificity, S_c, is the probability that for two randomly selected problem elements, there will be no collisions with solution elements across the entire system. This means that $S_c = \prod_{j=1}^{N}(1-P_{i_1 i_2 j})$, because for each solution element in the system, j, the probability that problem elements i_1 and i_2 will not both point to it is $(1-P_{i_1 i_2 j})$, and the probability that this will be the case for all j is the product of this probability across all j. We can rewrite S_c exactly as $S_c = \exp\left(\sum_{j=1}^{N}\ln(1-P_{i_1 i_2 j})\right) = \exp\left(\sum_{j=1}^{N}\left(-\sum_{k=1}^{\infty} {P_{i_1 i_2 j}}^k/k\right)\right)$ from the Maclaurin series expansion of $\ln(1-P_{i_1 i_2 j})$, which is always valid in this study because $P_{i_1 i_2 j} < 1$. Further, because $P_{i_1 i_2 j}$ is very small ($\ll 1$) in this work, due to the large problem spaces, the $k = 1$ term dominates, and

$$S_c \approx \exp\left(-\sum_{j=1}^{N} P_{i_1 i_2 j}\right) = e^{-P_c}, \tag{10}$$

since $P_c = \sum_{j=1}^{N} P_{i_1 i_2 j}$, as shown above. This is the general expression for the systemic OpS when the probability of each individual cross-reaction, $P_{i_1 i_2 j}$, is small.

In addition, when the systemic collision probability, P_c, itself, is small (as also is the case in the current work), then $e^{-P_c} \approx 1-P_c$ from the series expansion of the exponential, and hence $S_c \approx 1-P_c$, analogously to the single-element and average OpS expressions.

Hence, from Eq (9), for large M, we have

$$S_c \approx 1-\frac{N\langle D_j\rangle^2}{M^2}\left(\frac{\mathrm{Var}\,(D_j)}{\langle D_j\rangle^2}+1-\frac{1}{\langle D_j\rangle}\right) = 1-\frac{\langle D_i\rangle^2}{N}\left(\mathrm{Var}\,(R_j)+1\right) = 1-\frac{\langle D_i\rangle^2 K_c^\dagger}{N}, \tag{11}$$

provided $\frac{\langle D_i \rangle^2}{N}\left(\mathrm{Var}\left(R_j\right)+1\right)$ is small. For high average degeneracies ($\langle D_j \rangle \gg 1$), the distribution coefficients K_c and $K_c^{\dagger}$, are the factors by which the distribution raises the collision or cross-reaction probability relative to the minimum.

There are several points to note here:

- Unlike the average probability and OpS, $\langle P_j \rangle$ and $\langle S_j \rangle$, the systemic cross-reaction probability and OpS, P_c and S_c, depend on the variance[2] of the normalized solution element degeneracies, R_j.
- From Eq. (4), the probability of collisions (e.g., Ab cross-reactions), P_c, depends inversely on the repertoire size, N, and increases with the square of the average problem element (e.g., epitope) degeneracy, $\langle D_i \rangle$.
- P_c increases with increasing variance of the degeneracies, D_j, or normalized degeneracies, R_j; and for fixed $M,\ N$, $\langle D_i \rangle$, and $\langle D_j \rangle$, a zero variance in the degeneracies (i.e., $\mathrm{Var}\left(D_j\right)=\mathrm{Var}\left(R_j\right)=0$) minimizes P_c and maximizes systemic OpS. This is a so-called singular or spiked distribution, in which all $D_j=\langle D_j \rangle$, all $R_j=1$, and for which the distribution coefficient, $K_c=1-1/\langle D_j \rangle$.
- For high solution element degeneracies ($\langle D_j \rangle \gg 1$), as in the human Ab repertoire, the following are true:
  - From Eqs. (4), (9) or (11), for $M \gg 1$, as long as the D_j grow proportionately with M, the collision probability (and systemic OpS) is constant with increasing M.
  - The minimum value for the distribution coefficient is $K_{c,min} \approx 1$.
  - From Eqs. (4) and (11), for $M \gg 1$, the optimal (i.e., spiked) distribution of $\langle D_j \rangle$, which results in $K_c = K_{c,min} \approx 1$, also results in $P_{c,min} \approx \langle D_i \rangle^2/N$ and $s_{c,max} \approx 1-\langle D_i \rangle^2/N$.
- The Poisson distribution has the special property that $K_c=1$ in all cases, because its variance equals its mean, and hence $\mathrm{Var}\left(D_j\right)/\langle D_j \rangle^2 = 1/\langle D_j \rangle$.
- Increasing the overlap between Ab binding spaces by simply replacing paratopes (epitope binding sites) on particular antibodies to match those on others does not change the systemic Ab OpS (S_c, Eq. 11), since this process changes neither the distribution of Ab degeneracies –i.e., D_j's–the repertoire size, N, nor the average epitope degeneracy, $\langle D_i \rangle$.

Since the models are symmetric in problem and solution elements, the equivalent expressions for anticollisions–i.e., epitope complementarity to non-cognate antibodies– ($p_i, s_i, \langle p_i \rangle, \langle s_i \rangle, p_c$, and s_c) can be obtained from those above by swapping $N \leftrightarrow M$, and subscripts $j \leftrightarrow i, a \leftrightarrow c$. For example, the systemic OpS for anticollisions, $S_a = e^{-P_a}$, which is analogous to the case for collisions (Eq. 10). However, the above derivations involving $\langle P_j \rangle$ hold only when the solution element degeneracies, D_j, are strictly $\geq$1. The more general derivations for $\langle P_j \rangle$ (and P_c) that include solution elements having $D_j=0$ are given in Supplementary Material (3). Such extensions are important in the computation of $\langle P_i \rangle$ for anticollisions because the D_i's for epitopes are small, with $\langle D_i \rangle$ very likely less than one, which means the $\langle P_i \rangle$ and $\langle S_i \rangle$ derivations/formulas analogous to those above would not hold. By contrast, as shown in Supplementary Material (3), the anticollision equivalents of the P_c and S_c formulas (Eqs 11 and 4) are unchanged whether the $D_j=0$ terms are included or not and, thus, hold for any positive real D_i.

[2] Strictly, there are higher-order terms, because a given problem element can collide with two or more non-cognate elements (a single antibody can be complementary to two or more non-cognate epitopes), but those contributions vary as $\approx m/N^{m+1}$, for m collisions per solution element, so they are small compared to the pairwise terms ($m=1$) and can be safely ignored for large N.

6.2.4 Considering two subpopulations

If the number of solution elements in two populations having degeneracies D_{j_1} and D_{j_2} are n_1 and n_2, respectively, then $\mathrm{Var}\left(R_j\right) = \frac{n_1 n_2 (D_{j_1} - D_{j_2})^2}{\left(n_1 D_{j_1} + n_2 D_{j_2}\right)^2}$. This quantity is minimized when either n_1 or n_2 is zero or when $D_{j_1} = D_{j_2}$, which are conditions that imply a singular distribution. Hence, in cases where $\{n_1, n_2\} \neq 0$ and $D_{j_1} \neq D_{j_2}$, the variance will be above the minimum, implying a sub-maximal OpS.

6.3 The AEIP model: details and approximations

As mentioned in Methods (Section 2.3.1) the form of the AEIP model is

$$P(\varepsilon, m, n, N), = S_\varepsilon C_n T_1 T_2, \tag{3}$$

where $S_\varepsilon = \frac{\varepsilon!}{(\varepsilon - m)!}$, $C_n = \frac{n!}{(n-m)!m!}$, $T_1 = \frac{(N-n)!}{(N-n-\varepsilon+m)!}$, $T_2 = \frac{(N-\varepsilon)!}{N!}$,

where m is the number of Ab-Ag interactions, or matches, per antigen. The first term gives the permutations of matching epitopes, the second the combinations of the selected antibodies, and the third term (T_1) the permutations of non-matching epitopes. The last term (T_2) normalizes the result. Notably, the model is symmetric in the number of epitopes per Ag (ε) and the number of selected antibodies (n). Because of the large numbers involved in the factorials, to reduce numerical error in the calculations, we compute the log of the above expression as

$$\ln(P) = \sum_{k=\varepsilon-m+1}^{\varepsilon} \ln(k) + \sum_{k=n-m+1}^{n} \ln(k) - \sum_{k=1}^{m} \ln(k) - \sum_{k=N-\varepsilon+1}^{N} \ln(k) + \sum_{k=N-\varepsilon-n+m+1}^{N-n} \ln(k) \tag{12}$$

and then exponentiate the result.

The exact ratio of the probabilities of m matches to $m+1$ matches is $\frac{P(m)}{P(m+1)} = \frac{(m+1)(N-n-\varepsilon+m+1)}{(n-m)(\varepsilon-m)}$, and for $N \gg \{n,\ \varepsilon, m\}$, this is approximately

$$\frac{P(m)}{P(m+1)} \approx \frac{(m+1)N}{(n-m)(\varepsilon-m)}. \tag{13}$$

We can derive some simplifying approximations as follows. For large N,

$$T_1 \approx (N-n)^{\varepsilon-m+1} \tag{14}$$

and

$$T_2 \approx N^{-(\varepsilon+1)},$$

and hence

$$P(\varepsilon, m, n, N), \simeq S_\varepsilon C_n (N-n)^{\varepsilon-m+1} / N^{\varepsilon+1} = S_\varepsilon C_n \left(1 - \frac{n}{N}\right)^{\varepsilon-m+1} / N^m. \tag{15}$$

For $N \gg n(\varepsilon - m + 1)$, from Eq. (14), $T_1 \approx N^{\varepsilon-m+1}\left(1 - \frac{n(\varepsilon-m+1)}{N}\right)$, so that

$$P(\varepsilon, m, n, N), \approx S_\varepsilon C_n \left(1 - \frac{n(\varepsilon - m + 1)}{N}\right) / N^m. \tag{16}$$

For $N \gg n(\varepsilon - m + 1)$ and $m > 0$, the smaller term in parentheses can be ignored, and $T_1T_2 \approx 1/N^m$, so that the probability simplifies to

$$P(\varepsilon, m, n, N), \approx \frac{S_\varepsilon C_n}{N^m}, \tag{17}$$

as mentioned in the main text.

It can be shown that the expectation value for the number of epitope matches per antigen $\langle m \rangle$ is:

$$\langle m \rangle = \sum_{m=0}^{\varepsilon} mp = \varepsilon n/N. \tag{18}$$

Hence, the overall chances for interactions or matches grow slowly (linearly) with the number of epitopes or tested antibodies, for a fixed repertoire size (N).[3]

Separately, using Stirling's approximation, $n! = \sqrt{2\pi n}(n/e)^n$, we can show that $T_1 \approx \frac{e^{m-\varepsilon}(N-n)^{(N-n+1/2)}}{(N-n-\varepsilon+m)^{(N-n-\varepsilon+m+1/2)}}$ and $T_2 \approx \frac{e^{\varepsilon}(N-\varepsilon)^{(N-\varepsilon+1/2)}}{N^{N+1/2}}$, so that the probability of a cross-reactive match is

$$P(\varepsilon, m, n, N) \approx S_\varepsilon C_n \frac{e^m (N-n)^{\left(N-n+\frac{1}{2}\right)} (N-\varepsilon)^{\left(N-\varepsilon+\frac{1}{2}\right)}}{(N-n-\varepsilon+m)^{\left(N-n-\varepsilon+m+\frac{1}{2}\right)} N^{N+\frac{1}{2}}}. \tag{19}$$

For computational purposes, we again take the log of the equation and transform it (exactly) to obtain a form which helps avoid subtraction of large numbers and the accompanying precision-related errors:

$$\ln(P) = \ln(S_\varepsilon) + \ln(C_n) + m - m\ln(N) + N\left(\left(1 - \frac{2\varepsilon - 1}{2N}\right)\ln\left(1 - \frac{\varepsilon}{N}\right) + \left(1 - \frac{2n-1}{2N}\right)\ln\left(1 - \frac{n}{N}\right) - \left(1 - \frac{2(\varepsilon + n - m) - 1}{2N}\right)\ln\left(1 - \frac{\varepsilon + n - m}{N}\right)\right) \tag{20}$$

where $N > \varepsilon, n$, and $N > n + \varepsilon - m$, and then we exponentiate the result.

For large ε or n, the log of the falling factorials in $\ln(S_\varepsilon)$ or $\ln(C_n)$ can be similarly approximated as $\ln(n!/(n-m)!) \approx -(n-m+1)\ln\left(1 - \frac{m}{n}\right) + m\ (\ln(n) - 1)$, $n > m$.

6.4 Various other distribution coefficients

The variance of scaled chi distributions (e.g., the Rayleigh, Maxwell-Boltzmann, and higher-degree distributions, described in Supplementary Material 8), which are related to the Gaussian distribution, is bounded above by $\langle D \rangle^2(\pi - 2)/2$, where $\langle D \rangle$ is the distribution mean (106). Hence, for solution element degeneracies, D_j, conforming to these distributions, we can use Eq. (4) to set an upper bound for the maximal probability of systemic cross-reactivity as $P_{c,max} \approx \frac{1.57\langle D_i \rangle^2}{N}$, assuming large $\langle D_j \rangle$. For a uniform distribution of Ab degeneracies (see Figure 6, and Table 4), which is a limiting form of unimodal distribution, $\mathrm{Var}(R_j) = 1/3$, and hence $K_c^\dagger = 4/3$ and $P_c \approx \frac{1.333\langle D_i \rangle^2}{N}$. Lastly, Poisson always

[3] As an aside, if we introduce non-interacting epitopes ($D_i = 0$) into the model, so that $\langle D_i \rangle < 1$, then the number of matches will, on average, scale with it, so that $\langle m \rangle = \langle D_i \rangle \varepsilon n/N$. Further, if we select the entire repertoire, then, $\langle m \rangle = \langle D_i \rangle \varepsilon$ and $\langle D_i \rangle = \langle m \rangle/\varepsilon$, for fixed ε and $D_i < 1$.

have a distribution coefficient of $K_c = 1$ and $K_c^\dagger \approx 1 + 1/\langle D_j \rangle$ so that they (nearly) optimize OpS by approximating the distribution coefficient of the spiked distribution, particularly at high $\langle D_j \rangle$. [4]

As described in Section 6.2.4 above, relative to a spiked distribution of Ab degeneracies, systems having two sub-populations of antibodies with different degeneracies always have a higher P_c, for constant N. The type of distribution resulting in the lowest systemic OpS possible is one in which the total binding space is predominantly represented by a small group of n_2 antibodies that have very high degeneracies, D_{j_2}, while the great majority of antibodies (n_1) contribute relatively very little to the total binding space, such that $D_{j_2} \gg D_{j_1}$ and $n_2 D_{j_2} \gg n_1 D_{j_1}$. In that case, the variance in the normalized degeneracies reduces very approximately to $\mathrm{Var}\,(R_j) \approx \frac{n_1 n_2 (D_{j_2})^2}{\left(n_2 D_{j_2}\right)^2} = \frac{n_1}{n_2}$, which is roughly equal to the high-mean distribution coefficient, $K_c^\dagger = \frac{n_1}{n_2} + 1$, for $n_1 \gg n_2$. Assume, for example, that the human repertoire has $N = 10^{10}$ antibodies. If 10^7 highly degenerate antibodies made the predominant contribution to the binding space, while the others had a greatly reduced binding space, then $K_c^\dagger \approx 1000$, and the probability of a cross-reaction would increase roughly 1000-fold relative to that of the optimal configuration, and the OpS of the system would be reduced from the optimal 1-10^{10} to $\approx$1-10^7. See Figures 5 and 6.

[4] Since Poisson distributions are also a limiting form of Gaussians, i.e., when $\sigma^2 \to \mu$ at high means, Gaussian distributions over positive support with high means and σ^2 less than $\approx \mu$ also tend to optimize OpS.
