# Supplementary material for: Antibodies and cryptographic hash functions: quantifying the specificity paradox
Source: Front Immunol. 2025 Nov 5;16:1585421. doi: 10.3389/fimmu.2025.1585421 (PMC12627013; doi:10.3389/fimmu.2025.1585421)
Supplement: Supplementary file 2 [file DataSheet2.pdf]

Antibodies and cryptographic hash functions: quantifying the specificity paradox: Supplementary Material

[1] *Department of Chemistry and Chemical Biology, Harvard University, Cambridge, MA 02132,* [2] *Harvard Medical School, Boston, MA 02115*

Correspondence*:
petrella@fas.harvard.edu, robertjpetrella@yahoo.com

1 SUPPLEMENTARY TABLES AND FIGURE

1.1 Supplementary Figure

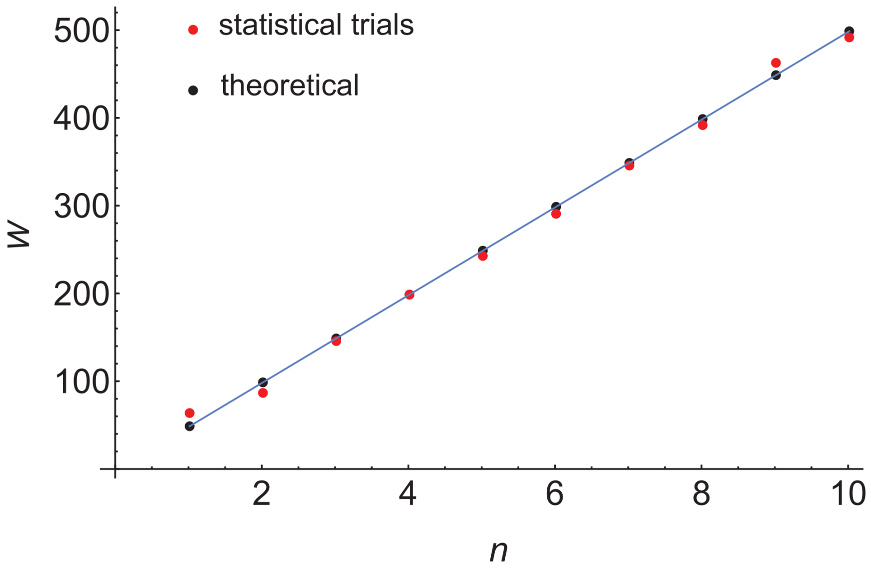

Figure 1. Plot of the number of antigens, W, cross-reacting exactly once as a function of the number of selected antibodies they are tested against, n. The repertoire size is fixed at 10 million, and the n antibodies are tested against 100 million randomly selected antigens, each having 5 epitopes. The red dots are the trial (simulation) results, while the black dots are the theoretical results, which are nearly, but not exactly, integral values. The blue line is a least-squares-fit of the theoretical results. The dependence of cross-reactivity on the number of available epitopes per antigen, here fixed at $\varepsilon = 5$, is similarly linear (not shown).

1.2 Supplementary tables: case examples of cross-reactive probabilities, including theoretical approximations

As described in Methods (Section 2.3), the AEIP model was developed to predict the average number of cross-reactive "matches" (complementarities) of epitopes on a number of randomly selected antigens to a set of antibodies that have been selected randomly from a larger Ab pool.

Table 1 shows the number of single-epitope cross reactions under varying repertoire size (N) with the other parameters fixed.

Table 2 shows the number of single-epitope cross reactions under varying number of test antibodies (n), with fixed repertoire size and other parameters fixed.

N	numerical	exact	$\Delta\%$	Δappr$_1$ %	Δappr$_2$ %	Δappr$_4$ %	ΔStirling's %
**100**	33946511905	33939091100.358	0.0219	47.3227	-26.3386	-13.0074	-8.574E-05
**1000**	4822094044	4821976189.512	0.0024	3.6919	-1.4927	-1.3900	-1.732E-05
**10^4**	498833733	498201979.622	0.1268	0.3609	-0.1409	-0.1399	-1.287E-05
**10^5**	50104527	49982001.980	0.2451	0.0360	-0.0140	-0.0140	3.170E-05
**10^6**	4977927	4999820.002	-0.4379	0.0036	-0.0014	-0.0014	-1.919E-05
**10^7**	496628	499998.200	-0.6740	3.600E-04	-1.400E-04	-1.400E-04	2.535E-05
**10^8**	49463	49999.982	-1.0740	3.600E-05	-1.400E-05	-1.400E-05	-2.497E-05
**10^9**	5241	5000.000	4.8200	3.600E-06	-1.400E-06	-1.400E-06	-7.363E-05
**10^{10}**	519	500.000	3.8000	3.600E-07	-1.400E-07	-1.400E-07	5.512E-05

Table S1. The number of single-epitope cross-reactive matches (i.e., $m = 1$) between 100 billion antigens and a set of 10 antibodies selected randomly from repertoires of varying sizes. The column with heading "***N***" lists the size of the Ab repertoire. The 2nd and 3rd columns give the number of antigens cross-reacting with one Ab according to either the phenomenological simulations ("**numerical**"), or the exact model results ("**exact**"), which equal AP, where P is the probability of a match derived from the theoretical estimates (Eq. 12) and A is the number of antigens tested (here, 100 billion). The "$\Delta\%$" column shows the percent difference between the numerical and exact results. The final 4 columns show the percent differences between four approximations to the AEIP model and the exact results: Δ**appr1%**: $P = S_\varepsilon C_n / N^m$, from Eq. (17); Δ**appr2%**: $P = S_\varepsilon C_n \left(1 - \frac{(\varepsilon - m + 1)n}{N}\right) / N^m$, from Eq. (16); Δ**appr3 %**: $P = \frac{S_\mathcal{E} C_n (N-n)^{\mathcal{E}-m+1}}{N^{(\mathcal{E}+1)}}$, from Eq. (15); Δ**Stirling's %**: formulation using the Stirling approximation, Eq. (20). The number of epitopes per antigen is fixed at 5 throughout. As the Ab pool becomes larger, antibody-antigen cross-reactive matches become less common, roughly in inverse proportion to the size of the repertoire. In these studies, the epitope degeneracy is fixed at $\langle D_i \rangle = 1$, so that epitope recognition is complete and non-overlapping. The Stirling's-approximation-based estimate is accurate to within $\pm 0.0001\%$ at all repertoire sizes, whereas the other approximations are far less accurate at smaller N and become increasingly more accurate with larger repertoire sizes. The differences between numerical and exact results ($\Delta\%$ column) are due to statistical error.

n	numer	exact	$\Delta\%$	Δappr$_1$ %	Δappr$_2$ %	Δappr$_4$ %	Δ Stirling's %	Δ exact w/o log %
1	65	50.000	30.000	1.279E-13	-5.000E-05	-5.000E-05	2.535E-05	8.274E-06
2	88	100.000	-12.000	4.000E-05	-6.000E-05	-6.000E-05	2.535E-05	1.275E-05
3	147	150.000	-2.000	8.000E-05	-7.000E-05	-7.000E-05	2.535E-05	3.009E-06
4	183	200.000	-8.500	1.200E-04	-8.000E-05	-8.000E-05	2.535E-05	7.482E-06
5	244	250.000	-2.400	1.600E-04	-9.000E-05	-9.000E-05	2.535E-05	-2.256E-06
6	292	299.999	-2.666	2.000E-04	-1.000E-04	-1.000E-04	2.535E-05	2.217E-06
7	347	349.999	-0.857	2.400E-04	-1.100E-04	-1.100E-04	2.535E-05	6.689E-06
8	393	399.999	-1.750	2.800E-04	-1.200E-04	-1.200E-04	2.535E-05	4.057E-06
9	464	449.999	3.111	3.200E-04	-1.300E-04	-1.300E-04	2.535E-05	8.530E-06
10	493	498.998	-1.202	3.600E-04	-1.400E-04	-1.400E-04	2.535E-05	1.300E-05

Table S2. The number of antigens cross-reacting exactly once as a function of the number of selected antibodies they're tested against (***n***). The repertoire size is fixed at 10 million, and the *n* antibodies are tested against 100 million randomly selected antigens, each having 5 epitopes. The final column gives the percent differences between the logarithmic form of the AEIP model (Eq. 12) and the non-log form, which are essentially precision-related errors. The rest of the column headings are as in Table S1 above. Interestingly the % error in the Stirling's approximation-based estimate (relative to log-form exact) is constant for values of *n* in this range, to within about 10^{-12}.

2 GLOSSARY

absolute specificity–a one-to-one relationship of two elements in an ordered pair or set of ordered pairs. The ability of a system to associate each input or problem element with a distinct output or solution element. In the case of antibody specificity for antigens, it refers to monospecificity.

adaptive immune system–that part of the immune system that responds to antigens by producing antibodies and activating T-cells directed against them, as distinct from the innate immune system. Hence, it includes both humoral (B-cell/antibody) and cell-mediated (T-cell) responses.

affinity maturation–that part of adaptive immunity by which antibodies are selected for their binding affinity to a particular antigen, and then amplified, mutated, and re-selected in a cyclical manner.

antibody–a molecule (immunoglobulin) produced by specialized B cell descendants to bind to a particular antigen.

anticollision–the relation of two distinct solution elements to the same problem element–for example, an epitope with binding complementarity to an antibody other than its cognate antibody, or (hypothetically) a single digital message hashing to two different digests.

antigen–a molecule, molecular fragment, or a collection thereof, that is recognized by the adaptive immune system.

antigenic totality–the postulate that (human) immune recognition for antigen-sized molecular structures is complete within a given individual's repertoire. The weak form is that every antigen contains at least one element in the set of all recognized epitopes, Φ_H. A stronger form is that every antigen contains multiple elements in Φ_H.

attack–in cryptanalysis, an attempt to compromise the security of a cryptographic protocol by exploiting particular vulnerabilities.

avalanche effect–the generation of a large change in the output or image of a relation from a small change in the input or preimage.

average multiplicity (of a relation)–the average number of elements in the image (solution domain) that are associated with each element in the preimage.

B cell–a type of immune cell (lymphocyte) that has antibodies as surface receptors and whose descendants (plasma cells) produce antibodies.

binding space–the set of all distinct, complementary antibody-epitope pairs.

bit–the smallest unit of digital information, taking the value of either 1 or 0; binary digit.

byte–a group of bits operated on as a unit.

chemical (epitope) binding space–the set of molecules or molecular fragments to which a molecule (antibody) can bind.

chemical space–most generally, the set of all possible molecular compounds in all their possible molecular conformations. Here, it is used to refer to the set of possible compounds or molecular fragments that are in the proper size range for antigens or haptens.

cognate– in reference to of an ordered pair of elements, having a uniquely paired relationship (one-to-one and onto) that implies causality or some other primary association. In immunology, it describes the relationship between an antigen and an antibody resulting from an immune response.

cognate subset– a subset of a relation in which all elements are cognate pairs. In immunity, a set of cognate epitope-antibody or antigen-antibody pairs.

collision–in cryptography, the mapping of two files to the same hash value, often used in the context of a large, pairwise file search. Here, it is defined as the mapping of a "non-cognate" file – a file that was not the originating file–to a target hash value. In cryptography, this file is often called a *second preimage*.

collision resistance–the difficulty of finding collisions for a given hash function.

complementary–appearing in the same ordered pair of elements in a relation, without regard to whether or not the elements are cognate. For example, an antibody can have multiple complementary antigens or epitopes. In the immunological literature, the term is sometimes used synonymously with "cognate"–i.e., corresponding to the cognate partner in an antibody-epitope pair.

coverage fraction–the fraction of the domain of a relation (problem domain) from which elements point to at least one element in the codomain (solution domain).

cross-reactivity–the complementarity of an antibody to a non-cognate epitope, or *vice versa*.

cryptanalysis–the study of breaking codes or decrypting information.

cryptographic hash function (or algorithm)–a function that takes a digital input of arbitrary length and returns an alphanumeric code of fixed length, a.k.a hash value or message digest.

cryptography–the study of encoding or encrypting information, usually for security purposes.

cryptology–the study of cryptanalysis and cryptography.

degeneracy–here, the number of distinct partner elements to which an element is complementary under a relation, even if zero. Equivalently, the number of unique problem/solution element pairs in which an element participates. With respect to antibodies, it is the number of distinct epitopes that a given antibody

can recognize (i.e., bind), and *vice versa* with respect to epitopes. In cryptography, it is the number of distinct digital files that are associated with the same hash value or digest, and *vice versa* with respect to files.

digest–the output of a hash function. Message digest. Hash value.

distribution coefficient–for large distribution means (e.g., $\langle D_j \rangle \gg 1$)), this is the factor by which the form of the distribution of the solution element degeneracies increases the probability of collisions relative to the optimal case, given problem and solution spaces of fixed size and a fixed average problem element degeneracy; or *vice versa* with respect to solution and problem elements.

epitope–that portion of a molecule, molecular fragment, or set of fragments (e.g., a set of portions of amino acids) in a particular 3-dimensional conformation, allowing for local fluctuations, that is involved in close interactions with an antibody.

fiber size–in set theory, the number of (problem) domain elements pointing to a given codomain (solution domain) element under a relation. Preimage cardinality of an individual image element.

file–in this work, a string or series of alphanumeric digital characters and spaces, usually conveying information such as a series of financial transactions. Digital file. Message.

germline antibody–a member of the original set of primary antibodies in an individual, before somatic mutations occur in the periphery.

hash value–a string of alphanumeric characters, or a digital code, returned by a hash function that has been applied to a given electronic input file or message. Message digest. Digital fingerprint.

humoral immunity–that part of the immune response involving B-cells and antibodies.

image–the set of solution space or codomain elements that are mapped to by a relation, from preimage elements.

immune repertoire–the set of all distinct antibodies or cell receptors in an individual's adaptive immune system.

immunogen–an antigen that is capable of triggering an immune response.

message–(digital) file.

message digest–the alphanumeric code resulting from a cryptographic hash function's operation on a message. Hash value. Digital fingerprint.

monospecificity–in immunology, the (hypothetical) capacity of an antibody to recognize only a single antigen or epitope–namely, its cognate antigen.

multiplicity–the number of codomain (image) or solution domain elements to which a set of preimage element points under a relation. Also image cardinality. When referring to individual preimage elements, this is equal to the degeneracy, but the degeneracy is a property of all domain elements, not just the preimage.

multispecific–in reference to a relation: being many-to-one. Each problem element points to multiple solution elements. In reference to a solution element: complementary to multiple problem elements, or having a degeneracy greater than 1. For example, a multispecific antibody can recognize multiple antigens.

non-cognate–in reference to an ordered pair: lying outside the primary or cognate subset of order pairs in a relation. In reference to an individual element: not the cognate partner. Participating in a non-cognate

subset. For example, a non-cognate antibody to an epitope is one that the epitope did not elicit. In this work, the term also refers to the relationship between a hash value and a digital file or message that was not its original preimage.

non-cognate degeneracy–the number of non-cognate partner elements to which a problem or solution element is complementary.

non-specific– in the immunologic and biochemical literature, this often refers to *non-selective*–i.e., not exhibiting the primary or intended relationship between problem and solution elements. It can also mean multispecific–i.e., having a many-to-one relationship. Also, the inability of a system to recognize at least some non-cognate or non-primary partner elements in a relation as such.

normalized degeneracy–the degeneracy divided by the mean degeneracy of a set of elements.

operational specificity (OpS)– in general, the unlikelihood of random cross-reaction or collision. For individual partner elements and their averages, it is generally $S = 1–P$, where P is the probability that a randomly selected input element will result in a non-cognate pairing–for example, that a randomly chosen epitope will bind a given antibody, or, in cryptography, that a randomly chosen file will hash to a given target digest. The *systemic OpS* is $S \approx e^{-P}$, where P is the probability (or number) of random cross-reactions across the entire system, provided the probabilities of individual cross-reactions are small $(\ll 1)$. When P, itself, is small, systemic OpS is $S \approx 1 - P$.

pigeonhole principle–the fact that a set of M domain elements cannot be mapped uniquely to a set of N codomain elements, if $M > N$. More generally, if more than kN objects are placed into N boxes, then at least one box must contain more than k objects.

polyclonal response–the production of multiple antibodies by the adaptive immune response against an antigen, each directed toward a different epitope.

polyreactive–polyspecific. Often used in the context of weak interactions.

polyspecific–complementary to multiple antigens or epitopes (said of antibodies or immune cell receptors). Multispecific.

preimage–the subset of (problem) domain elements under a relation that point to at least one element in the codomain (i.e., the solution domain).

preimage cardinality–the number of elements in the (problem) domain associated with a set of codomain (solution domain) elements under a relation. For individual image elements, this is equal to their degeneracy.

primary antibodies–the antibodies and corresponding receptors that exist in the adaptive immune system prior to challenge by an antigen and the triggering of affinity maturation. See also germline antibodies.

problem domain–the set of elements that contains those that are assigned to, or associated with, elements of a solution domain. Problem space.

random oracle– a theoretical black box responding to each unique input or problem element with a fixed result that is chosen according to a (pseudo)random, uniform distribution from its output or solution domain.

relation–a set of associations between problem and solution elements (ordered pairs) that is not necessarily a function.

relation size–the total number of distinct pairs of preimage and image elements in a relation. In adaptive immunity, it may refer to the size of the antibody-antigen binding space.

secure hash algorithm (SHA)–cryptographic hash function.

solution domain–the set of elements that contains those that are associated with problem elements by a relation. Solution space.

specificity–in general, the ability of a system to recognize a negative result (e.g., a non-cognate relation or the absence of medical condition) as such. In binary medical tests, for example, it is the number of true negative test results as a fraction of the number of actual negatives in the population. In the immunological literature, it sometimes refers to absolute specificity. The term is often used in an ill-defined context.

T cell–a type of immune cell (lymphocyte) that has antibody-like molecules as surface receptors and whose 'helper' subtype assists B cells in the adaptive immune response.

3 INCLUDING ELEMENTS OF DEGENERACY 0 IN COLLISIONS AND ANTICOLLISIONS.

3.1 Collisions

For systems in which there exist solution elements having $D_j = 0$ that need to be taken into account, e.g., when $\langle D_j \rangle$ is small or less than 1, the derivations are as follows.

The number of non-cognate problem elements complementary to a solution element, averaged across all solutions elements is

$$\langle {D_j}^* \rangle = \sum_{k=2}^{M} (k-1) L_k = \sum_{k=2}^{M} k L_k - \sum_{k=2}^{M} L_k, \tag{S1}$$

where L_k is the fraction of solution elements having $D_j = k$, and $1 < D_j < M$. Expanding,

$$\langle {D_j}^* \rangle = \sum_{k=0}^{M} k L_k - 1L_1 - 0L_0 - \left(\sum_{k=0}^{M} L_k - L_1 - L_0 \right) = \sum_{k=0}^{M} k L_k - \left(\sum_{k=0}^{M} L_k - L_0 \right),$$

as the L_1 terms cancel. And since $\sum_{k=0}^{M} k L_k = \langle D_j \rangle$ and $\sum_{k=0}^{M} L_k = 1$, it is true that $\langle {D_j}^* \rangle = \langle D_j \rangle - 1 + L_0$, and hence the average collision probability for an individual solution element is, then,

$$\langle P_j \rangle = \frac{\langle {D_j}^* \rangle}{M} = \frac{\langle D_j \rangle - 1 + L_0.}{M} \tag{S2}$$

This differs from the expression for $\langle P_j \rangle$ we derived previously for integral D_j strictly ≥ 1 (Eq. 7) in the Appendix (Section 8.2 of the main text) by the additional L_0 term, which is the fraction of elements having degeneracy 0.

Now, applying a similar treatment to the systemic solution element collision probability,

$$P_c = \sum_{k=2}^{M} \left(\left(\tfrac{k}{M}\right) \left(\tfrac{k-1}{M-1}\right) L_k \right) = \tfrac{N}{M(M-1)} \sum_{k=2}^{M} k(k-1) L_k = \tfrac{N}{M(M-1)} \left(\sum_{k=2}^{M} k^2 L_k - \sum_{k=2}^{M} k L_k \right) =$$

$$\tfrac{N}{M(M-1)} \left(\sum_{k=0}^{M} k^2 L_k - 0^2 L_0 - 1^2 L_1 - \left(\sum_{k=0}^{M} k L_k - 0L_0 - 1L_1 \right) \right).$$

Since the L_0 terms are 0 and the L_1 terms cancel, then,

$P_c = \frac{N}{M(M-1)}\left(\sum_{k=0}^{M} k^2 L_k - \sum_{k=0}^{M} k L_k\right) = \frac{N}{M(M-1)}\left(\sum_{i=1}^{N} {D_j}^2/N - \langle D_j \rangle\right)$,

and since $\mathrm{Var}\,(D_j) = \sum_{i=1}^{N} {D_j}^2/N - \langle D_j \rangle^2$,

$P_c = \frac{N}{M(M-1)}(\mathrm{Var}\,(D_j) + \langle D_j \rangle^2 - \langle D_j \rangle)$,

which is equivalent to the expression for P_c in the main text. Hence, we have confirmed this formulation holds for small as well as large values of D_j.

3.2 Anticollisions

For anticollisions, we simply swap i and j, as well as M and N (and subscripts a and c). For example, suppose a Poisson distribution of epitope degeneracies $L_k = \frac{e^{-\langle D_i \rangle} \langle D_i \rangle^k}{k!}$ with mean $\langle D_i \rangle$. Then, the average epitope probability of cross-reactivity to an antibody (i.e., the average anticollision probability) is:

$$\langle P_i \rangle = \frac{\langle D_i \rangle - 1 + L_0}{N} = \frac{\langle D_i \rangle - 1 + e^{-\langle D_i \rangle}}{N}. \tag{S3}$$

For small $\langle D_i \rangle$, by 2nd-order series expansion of the exponential, this is
$\langle P_i \rangle \approx \frac{1}{N}\left(\langle D_i \rangle - 1 + \left(1 - \langle D_i \rangle + \frac{\langle D_i \rangle^2}{2}\right)\right) = \langle D_i \rangle^2/2N$. For larger $\langle D_i \rangle$, $e^{-\langle D_i \rangle} \to 0$ and $\langle P_i \rangle$ thus approaches $(\langle D_i \rangle - 1)/N$, which is the expected result.

For the same distribution, the systemic anticollision (epitope cross-reaction) probability is

$$P_a = \frac{\langle D_j \rangle^2}{M} \frac{N}{(N-1)} \left(\frac{\mathrm{Var}\,(D_i)}{\langle D_i \rangle^2} + 1 - \frac{1}{\langle D_i \rangle}\right) =$$

$$\frac{\langle D_j \rangle^2}{M} \frac{N}{(N-1)} \left(\frac{\langle D_i \rangle}{\langle D_i \rangle^2} + 1 - \frac{1}{\langle D_i \rangle}\right) = \frac{\langle D_j \rangle^2}{M} \frac{N}{(N-1)} = \frac{\langle D_i \rangle^2 M}{N(N-1)}. \tag{S4}$$

which holds for any range of D_i.

4 PARAMETER ESTIMATES FOR THE CALCULATION OF ANTIBODY SELF-REACTIVITY

Estimate for the number of accessible self-antigens. The size of the human proteome has been the subject of much study and considerable debate, and theoretical estimates of its size range from 10,000 to millions. In contrast to the large number of antibodies/cell receptors in the human immune repertoire, there are fewer than 20,000 protein-coding human genes ((1)). As of June, 2023, the Human Protein Atlas contained data on 17,288 unique human proteins ((2, 3)), and as of 2022, the Human Proteome Project had catalogued over 18,000 of the predicted 19,778 human proteins ((4, 5)). However, less than half of protein species are outside of cells and therefore generally accessible to antibodies ((6, 2)). About 4400 canonical proteins have been found in human plasma ((7)). Immunoglobulins, themselves, can be antigens ((8)), but many of those species are present in very small numbers unless their production is stimulated ((9)). For the purposes of estimating an antibody's cross-reactivity to self, a fair, order-of-magnitude estimate for the number of self-antigens is 10,000.

Estimate for the average number of accessible epitopes per antigen, ϵ. The average human protein is composed of about 400-500 amino acids ((10, 11, 12)). This means it will have slightly less than that

number of epitope-length linear segments[1]. Discontinuous epitopes are generally thought to outnumber linear epitopes by a wide margin, perhaps 9 to 1 or more ((13, 14, 15)). A 2012 analysis indicated 12% of protein epitopes were composed of a single continuous segment, if a gap of up to 3 amino acids ((15)) is allowed. The ratios imply a theoretical upper bound for the total epitopes per protein of about 5000. However, in reality, not all of these epitopes will be exposed on the protein or cell surface or elicit a strong immune response. Epitope mapping studies have typically detected a handful to a few dozen immunodominant epitopes per protein ((16, 17, 18, 19, 20, 21, 22)). Some 1200 distinct monoclonal antibodies were raised against the protein BlyS from antibody phage display libraries derived from 80 people ((23)). Taken together, these data suggest a generous approximation of 1000 for ϵ.

5 PREDICTION OF DEGENERACY STATISTICS ACCORDING TO A TRUNCATED GAUSSIAN DISTRIBUTION.

For a Gaussian distribution of Ab degeneracies (D_j) truncated at $D_j = a$ on the left tail and $D_j = b$ on the right, the probability distribution function is

$$f\left(D_j\right) = \frac{e^{-(D_j-\mu)^2/2\sigma^2}}{\sigma\sqrt{\pi/2}\,h} dD_j, \tag{S5}$$

where $h = \mathrm{Erf}\left(\frac{\mu-a}{\sqrt{2}\sigma}\right) - \mathrm{Erf}\left(\frac{\mu-b}{\sqrt{2}\sigma}\right)$.

Then, the predicted (or expected) mean in the degeneracies is

$$\langle D_j\rangle_G = \int_a^b f(D_j)\cdot D_j\, dD_j = \mu + \sigma\sqrt{2/\pi}\left(E_a - E_b\right)/h, \tag{S6}$$

where $E_a = e^{-(\mu-a)^2/2\sigma^2}$ and $E_b = e^{-(\mu-b)^2/2\sigma^2}$.

The predicted variance in the degeneracies is

$$\mathrm{Var}\left(D_j\right)_G = \int_a^b f(D_j)\cdot {D_j}^2\, dD_j - \left(\int_a^b f(D_j)\cdot D_j\, dD_j\right)^2 =$$

$$\sigma^2 - \frac{2}{\pi}\left(\frac{\sigma}{h}\right)^2\left(E_a - E_b\right)^2 - \sqrt{\frac{2}{\pi}}\left(\frac{\sigma}{h}\right)\left(E_a(u-a) - E_b(u-b)\right), \tag{S7}$$

where, in this work, $a = D_{\mathrm{min}} - w/4$ and $b = D_{\mathrm{max}} - w/4$, D_{min} and D_{max} are the first and last non-empty bins in the distribution, and w is the bin width. The $w/4$ terms are used to partly compensate for discretization error.

The predicted rates of interaction were calculated as

$$rate_G = \left(\frac{\mathrm{Var}\left(D_j\right)_G}{{\langle D_j\rangle_G}^2} + 1\right)/N \tag{S8}$$

[1] A protein with L amino acids will have $L - L + 1$ linear epitopes of length L.

Preimage attack	
Given a target digest, find a corresponding message ((24)).	Given an antibody, find a complementary epitope ((25)).
2nd preimage attack	
Given a message and its digest, find a new message producing the same digest (2nd Preimage) ((26)).	Given an epitope and its cognate antibody, find a second (cross-reactive) complementary antibody ((27, 28)).
Length extension attack	
Given a message and its digest, extend the message and, starting from the original message, find a new digest ((29, 30)).	Given an epitope, extend it (i.e., enlarge it) and based on the cognate antibody structure, find a cross-reacting antibody.
Multicollision attack	
Find ϵ nested collisions and combine the messages in all possible ways to produce 2^n collisions ((31)).	Find an antibody that cross-reacts with n epitopes differing by small changes, so that as a byproduct the antibody also cross-reacts with other epitopes containing multiple combinations of the ϵ sets of changes.
Differential attack	
Given a message and its digest, perturb the message slightly to produce a collision ((32, 33, 34)).	Given an epitope and its cognate antibody, alter the epitope slightly (e.g., single or combinations of individual amino acid substitutions) so as to cross-react with the original antibody ((17)).
Correcting block attack	
Given a message and its digest, change a contiguous portion of the message (block) so as to generate a collision ((35)).	Given an epitope and its cognate antibody, alter a contiguous portion of the epitope to cross-react with the antibody ((36)).
Fixed point attack	
Given a message and its digest, find an extension to the message (chaining variable) that results in the same digest. Any arbitrary number of instances of the extension can be inserted to produce collisions.	Given an epitope and its cognate antibody, find a position in the epitope (or its extension) where changes–for example, variation in the amino acid sequence or chemical modifications at one or more positions–result in continued complementarity of the antibody.
Herding attack	
Given the initial portion of a message and a target digest, find the final portion of the message that results in the target by precomputing intermediate values colliding with the target ((37))	Given a portion of an epitope, find the remaining portion of the epitope that will bind a target antibody, by finding intermediate versions of the epitope that bind.

Table S3. List of several types of attacks on cryptographic hash functions (1st column) and analogous problems/procedures in experimental immunology (2nd column).

6 AN UPPER BOUND FOR LIFETIME ANTIGENIC EXPOSURES

It has been estimated that children are exposed to 2-6,000 antigens per day ((38)), and the maximal total antigen exposure from vaccines in children is about 6000 antigens by 3 months of age ((39)). For an upper bound estimate of total lifetime exposures, we can allow for 10,000 new antigen exposures a day, which includes the equivalent of receiving all of those vaccinations—but with different antigens each time—in addition to a large number of antigenically distinct environmental exposures, every day for an individual's lifetime. Over 100 years, that equals 365 million antigen exposures. Assuming 1000 epitopes per antigen, that's over 10^{10} distinct epitope lifetime exposures. Given that people tend to be exposed to the same antigens every day, that estimate is probably several orders of magnitude too high—it roughly equals or even exceeds the body's antibody repertoire—but it still represents only a tiny fraction of epitope space (at least 10^{83} distinct, antigen-sized molecules or molecular fragments).

7 ADDITIONAL PARALLELS BETWEEN FIELDS

Although a great deal of cryptanalysis is devoted to attacking the vulnerabilities of cryptographic algorithms and their components ((33, 31, 40, 37, 29, 41, 30)), ((34, 42, 43, 32, 44, 45)), the current secure state of functions such as SHA-256 means that calculating their inverse, i.e., constructing or designing files that will generate a particular target hash value, is impossible in practice. This is the case despite the very large number of possible alternative files that hash to each target. Hence, finding these digital files, or messages, in practical applications like Bitcoin mining or security protocols requires "brute force" methods: many different files are selected essentially at random and then hashed by the algorithm one-by-one to determine whether any of them result in the target digest. The inability to back-calculate messages resulting in an intended hash value target is called *preimage resistance* or *one-wayness* ((46)) and, along with collision resistance, is fundamental to the algorithms' utility ((24)). The immunological equivalent to this, designing novel antigens for a given, known antibody, though not trivial, is more computationally tractable, at least in the case of searching for structural homology in proteins ((47)), and can be accomplished experimentally, for example, with the use of combinatorial libraries created by phage display or transient co-transfection techniques ((48, 49, 50)).

Various other forms of attack that have been directed toward vulnerabilities of SHAs and their components suggest analogous problems and procedures in immunology; a number of these are listed in the Table S3. Lastly, the statistical ideas discussed in this work also have parallels in other fields. For example, in cosmology, they may help to explain the Fermi paradox ((51)), because extraterrestrial life may be abundant in absolute quantity but the size of the universe still renders it highly unlikely to coincide (or "collide") with a randomly chosen point in space and time–e.g., us, in the present day. Similarly, when two galaxies collide, the probability that any pair of constituent stars will collide is small ((52, 53)), despite the extremely large stellar populations.

8 FORMS OF CHI-DISTRIBUTIONS

The general form for the pdf of scaled chi-distributions is

$$f(D,\sigma,\widetilde{n}) = \frac{2\, e^{-D^2/2\sigma^2} D^{\widetilde{n}-1}}{\left(\sqrt{2}\sigma\right)^{\widetilde{n}} \Gamma\left(\widetilde{n}/2\right)} dD, \tag{S9}$$

where σ is the spread parameter and $\widetilde{n}$ is the number of degrees of freedom. For Rayleigh distributions, $\widetilde{n}$ = 2, and for Maxwell-Boltzmann distributions, $\widetilde{n}$ = 3.

Over the intervals $D \in [a, \infty)$ or $D \in [0, a)$, where $a \geq 0$, it can be shown that the maximal variance occurs over the interval $[0, \infty)$ and at $\widetilde{n} = 1$ and that it is equal to $\langle D\rangle^2(\pi - 2\ )/2 \approx 0.571\langle D\rangle^2$, where $\langle D\rangle$ is the mean of the distribution. ((54)).

REFERENCES

1 .Kim MS, Pinto SM, Getnet D, Nirujogi RS, Manda SS, Chaerkady R, et al. A draft map of the human proteome. *Nature* **509** (2014) 575–581. doi:10.1038/nature13302.

2 .Uhlén M, Fagerberg L, Hallström BM, Lindskog C, Oksvold P, Mardinoglu A, et al. Tissue-based map of the human proteome. *Science* **347** (2015) 1260419. doi:10.1126/science.1260419.

3 .The Human Protein Atlas Project. HPA–The open access resource for human proteins (2023). Available at https://www.proteinatlas.org.

4 .Legrain P, Aebersold R, Archakov A, Bairoch A, Bala K, Beretta L, et al. The human proteome project: current state and future direction. *Mol Cell Proteomics* **10** (2011) M111.009993. doi:10.1074/mcp.M111.009993.

5 .Human Proteome Organization. HPP progress to date (phase I) (2022). Available at: https://hupo.org/hpp-progress-to-date.

6 .Almén MS, Nordström KJV, Fredriksson R, Schiöth HB. Mapping the human membrane proteome: a majority of the human membrane proteins can be classified according to function and evolutionary origin. *BMC Biol* **7** (2009) 50. doi:10.1186/1741-7007-7-50.

7 .Deutsch EW, Omenn GS, Sun Z, Maes M, Pernemalm M, Palaniappan KK, et al. Advances and utility of the human plasma proteome. *Journal of Proteome Research* **20** (2021) 5241–5263. doi:10.1021/acs.jproteome.1c00657.

8 .Rodkey LS. Autoregulation of immune responses via idiotype network interactions. *Microbiol Rev* **44** (1980) 631–59. doi:10.1128/mr.44.4.631-659.1980.

9 .López-Requena A, Burrone OR, Cesco-Gaspere M. Idiotypes as immunogens: facing the challenge of inducing strong therapeutic immune responses against the variable region of immunoglobulins. *Frontiers in Oncology* **2** (2012). doi:10.3389/fonc.2012.00159.

10 .Skovgaard M, Jensen LJ, Brunak S, Ussery D, Krogh A. On the total number of genes and their length distribution in complete microbial genomes. *Trends Genet* **17** (2001) 425–8. doi:10.1016/s0168-9525(01)02372-1.

11 .Milo R, Phillips R. How big is the "average" protein? (2023). http://book.bionumbers.org/how-big-is-the-average-protein/.

12 .Tiessen A, Pérez-Rodríguez P, Delaye-Arredondo LJ. Mathematical modeling and comparison of protein size distribution in different plant, animal, fungal and microbial species reveals a negative correlation between protein size and protein number, thus providing insight into the evolution of proteomes. *BMC Research Notes* **5** (2012). doi:10.1186/1756-0500-5-85.

13 .Barlow DJ, Edwards MS, Thornton JM. Continuous and discontinuous protein antigenic determinants. *Nature* **322** (1986) 747–748. doi:10.1038/322747a0.

14 .Rubinstein ND, Mayrose I, Halperin D, Yekutieli D, Gershoni JM, Pupko T. Computational characterization of B-cell epitopes. *Molecular Immunology* **45** (2008) 3477–3489. doi:10.1016/j.molimm.2007.10.016.

15 .Sivalingam GN, Shepherd AJ. An analysis of B-cell epitope discontinuity. *Molecular Immunology* **51** (2012) 304–309. doi:10.1016/j.molimm.2012.03.030.

16 .McLane KE, Fritzen M, Wu X, Diethelm B, Maelicke A, Conti-Tronconi BM. Species- and subtype-specific recognition by antibody WF6 of a sequence segment forming an α-bungarotoxin binding site on the nicotinic acetylcholine receptor α subunit. *Journal of Receptor Research* **12** (1992) 299–321. doi:10.3109/10799899209074798.

17 .Buus S, Rockberg J, Forsström B, Nilsson P, Uhlen M, Schafer-Nielsen C. High-resolution mapping of linear antibody epitopes using ultrahigh-density peptide microarrays. *Mol Cell Proteomics* **11** (2012) 1790–1800. doi:10.1074/mcp.m112.020800.

18 .Wine Y, Boutz DR, Lavinder JJ, Miklos AE, Hughes RA, Hoi KH, et al. Molecular deconvolution of the monoclonal antibodies that comprise the polyclonal serum response. *Proc. Natl. Acad. Sci* **110** (2013) 2993–2998. doi:10.1073/pnas.1213737110.

19 .Molek P, Bratkovič T. Epitope mapping of mono- and polyclonal antibodies by screening phage-displayed random peptide libraries. *Acta Chim. Slov* **63** (2016) 914–919. doi:10.17344/acsi.2016.2458.

20 .Naqid IA, Owen JP, Maddison BC, Spiliotopoulos A, Emes RD, Warry A, et al. Mapping polyclonal antibody responses to bacterial infection using next generation phage display. *Sci Rep* **6** (2016) 24232. doi:10.1038/srep24232.

21 .Nogal B, Bianchi M, Cottrell CA, Kirchdoerfer RN, Sewall LM, Turner HL, et al. Mapping polyclonal antibody responses in non-human primates vaccinated with HIV Env trimer subunit vaccines. *Cell Reports* **30** (2020) 3755–3765.e7. doi:10.1016/j.celrep.2020.02.061.

22 .Lu S, Xie Xx, Zhao L, Wang B, Zhu J, Yang Tr, et al. The immunodominant and neutralization linear epitopes for SARS-CoV-2. *Cell Reports* **34** (2021) 108666. doi:10.1016/j.celrep.2020.108666.

23 .Edwards BM, Barash SC, Main SH, Choi GH, Minter R, Ullrich S, et al. The remarkable flexibility of the human antibody repertoire; isolation of over one thousand different antibodies to a single protein, BLyS. *Journal of Molecular Biology* **334** (2003) 103–118. doi:10.1016/j.jmb.2003.09.054.

24 .Rogaway P, Shrimpton T. Cryptographic hash-function basics: Definitions, implications, and separations for preimage resistance, second-preimage resistance, and collision resistance. Roy B, Meier W, editors, *Fast Software Encryption* (Springer Berlin Heidelberg) (2004), 371–388.

25 .Lemass D, O'Kennedy R, Kijanka G. Referencing cross-reactivity of detection antibodies for protein array experiments. *F1000Research* **5** (2017) 73. doi:10.12688/f1000research.7668.2.

26 .Kelsey J, Schneier B. Second preimages on *n*-bit hash functions for much less than 2^n work. *IACR Cryptology ePrint Archive* **2004** (2004) 304.

27 .Tao YL, Cheng XJ, Fu YF, Tsukamoto H, Yoshihara E, Tach ibana H. Modification of a human monoclonal antibody Fab fragment specific for *Plasmodium falciparum* 19-kDa C-terminal merozoite surface protein 1 by site-directed mutagenesis. *Parasitology Research* **103** (2008) 429–433. doi:10.1007/s00436-008-0994-4.

28 .Bostrom J, Yu SF, Kan D, Appleton BA, Lee CV, Billeci K, et al. Variants of the antibody herceptin that interact with HER2 and VEGF at the antigen binding site. *Science* **323** (2009) 1610–4. doi:10.1126/science.1165480.

29 .Dodis Y, Ristenpart T, Shrimpton T. Salvaging Merkle-Damgård for practical applications. Joux A, editor, *Advances in Cryptology - EUROCRYPT 2009, Lecture Notes in Computer Science* (Springer Berlin Heidelberg) (2009), vol. 5479, 371–388. doi:10.1007/978-3-642-01001-9_22.

30 .Gligoroski D. Length extension attack on narrow-pipe SHA-3 candidates. Gusev P M Mitrevski, editor, *ICT Innovations 2010* (Springer Berlin Heidelberg) (2010), 5–10. doi:10.1007/978-3-642-19325-5_2.

31 .Joux A. Multicollisions in iterated hash functions. application to cascaded constructions. Franklin M, editor, *Advances in Cryptology – CRYPTO 2004. Lecture Notes in Computer Science.* (Springer Berlin Heidelberg) (2004), vol. 3152, 306–316. doi:10.1007/978-3-540-28628-8_19.

32 .Dinur I, Dunkelman, O A Shamir. Collision attacks on up to 5 rounds of SHA-3 using generalized internal differentials. Moriai S, editor, *Fast Software Encryption* (Springer Berlin Heidelberg) (2014), vol. 8424, 219–240. doi:10.1007/978-3-662-43933-3_12.

33 .Biham E. On the applicability of differential cryptanalysis to hash functions. *E.I.S.S. Workshop on Cryptographic Hash Functions* (Oberwolfach(D)) (1992).

34 .Lamberger M, Mendel F. Higher-order differential attack on reduced SHA-256. *IACR Cryptol. EPrint Arch* (2011) 37.

35 .Tiwari H, Asawa K. Enhancing the security level of SHA-1 by replacing the MD paradigm. *J. Comput. Inf. Technol.* **21** (2013) 223–233.

36 .van't Hof W, Driedijk P, van den Berg M, Beck-Sickinger A, Jung G, Aalberse R. Epitope mapping of the *Dermatophagoides pteronyssinus* house dust mite major allergen Der p II using overlapping synthetic peptides. *Mol Immunology* **28** (1991) 1225–32. doi:10.1007/s00436-008-0994-4.

37 .Kelsey J, Kohno T. Herding hash functions and the Nostradamus attack. Vaudenay S, editor, *Advances in Cryptology - EUROCRYPT 2006. Lecture Notes in Computer Science.* (Springer Berlin Heidelberg) (2006), vol. 4004, 183–200. doi:10.1007/11761679_12.

38 .American Academy of Pediatrics. Child immunization schedule: Why is it like that? (2018). Available at: https://www.healthychildren.org/English/safety-prevention/immunizations/Pages/Child-Immunization-Schedule-Why-Is-It-Like-That.aspx.

39 .DeStefano F, Price CS, Weintraub ES. Increasing exposure to antibody-stimulating proteins and polysaccharides in vaccines is not associated with risk of autism. *The Journal of Pediatrics* **163** (2013) 561–567. doi:10.1016/j.jpeds.2013.02.001.

40 .Wang X, Yin Y, Yu H. Finding collisions in the full SHA-1. Shoup V, editor, *Advances in Cryptology – CRYPTO 2005. Lecture Notes in Computer Science.* (Springer Berlin Heidelberg) (2005), vol. 3621, 17–36. doi:https://doi.org/10.1007/11535218_2.

41 .Kuwakado H, Hirose S. Pseudorandom-function property of the step-reduced compression functions of SHA-256 and SHA-512. Chung KI, Sohn K, Yung M, editors, *Information Security Applications. Lecture Notes in Computer Science.* (Springer Berlin Heidelberg) (2008), vol. 5379, 174–189. doi:10.1007/978-3-642-00306-6_13.

42 .Naya-Plasencia M, Röck A, Meier W. Practical analysis of reduced-round Keccak. Bernstein D, Chatterjee S, editors, *Progress in Cryptology – INDOCRYPT 2011* (Springer Berlin Heidelberg) (2011), vol. 7107, 236–254. doi:10.1007/978-3-642-25578-6_18.

43 .Kölbl S, Mendel F, Nad T, Schläffer M. Differential cryptanalysis of Keccak variants. Stam M, editor, *Cryptography and Coding, IMACC 2013. Lecture Notes in Computer Science.* (Springer Berlin Heidelberg) (2013), vol. 8308, 141–157. doi:10.1007/978-3-642-45239-0_9.

44 .Dinur I, Morawiecki P, Pieprzyk J, Srebrny M, Straus M. Cube attacks and cube-attack-like cryptanalysis on the round-reduced Keccak sponge function. Oswald E, Fischlin M, editors, *Advances in Cryptology – EUROCRYPT 2015* (Springer Berlin Heidelberg) (2015), vol. 9056, 733–761. doi:10.1007/978-3-662-46800-5_28.

45 .YS S, Windarta S. Parallel algorithm to find collision in Merkle-Damgard construction with fixed point for 2(n/2)/k work. *International Seminar on Application for Technology of Information and Communication (ISemantic)* (IEEE) (2016). doi:DOI:10.1109/ISEMANTIC.2016.7873801.

46 .Merkle R. One way hash functions and DES. Brassard G, editor, *Advances in Cryptology — CRYPTO' 89 Proceedings* (Springer New York) (1990), 428–446. doi:10.1007/0-387-34805-0_40.

47 .McGill JR, Lagassé HAD, Hernandez N, Hopkins L, Jankowski W, McCormick Q, et al. A structural homology approach to identify potential cross-reactive antibody responses following SARS-CoV-2 infection. *Scientific Reports* **12** (2022) 11388. doi:10.1038/s41598-022-15225-3.

48 .Jiang B, Liu W, Qu H, Meng L, Song S, Ouyang T, et al. A novel peptide isolated from a phage display peptide library with trastuzumab can mimic antigen epitope of HER-2. *J Biol Chem* **280** (2005) 4656–62. doi:10.1074/jbc.M411047200.

49 .Mayrose I, Shlomi T, Rubinstein ND, Gershoni JM, Ruppin E, Sharan R, et al. Epitope mapping using combinatorial phage-display libraries: a graph-based algorithm. *Nucleic Acids Research* **35** (2006) 69–78. doi:10.1093/nar/gkl975.

50 .McLellan JS, Chen M, Joyce MG, Sastry M, Stewart-Jones GBE, Yang Y, et al. Structure-based design of a fusion glycoprotein vaccine for Respiratory Syncytial Virus. *Science* **342** (2013) 592–598. doi:10.1126/science.1243283.

51 .Jones E. Where is everybody? *Phys. Today* **38** (1985) 11–13.

52 .Binney S Jand Tremaine. *Collisions and Encounters of Stellar Systems* (Princeton: Princeton University Press), book section 8. Second edn. (2008).

53 .Frank R. Why stars never collide and galaxies always do (2023). https://bigthink.com/13-8/why-galaxies-collide/.

54 .Petrella R. The maximal variance of unilaterally truncated Gaussian and chi distributions. *In submission* (2025).
